# Convergence of location, direction, and theta in the rat anteroventral thalamic nucleus

**DOI:** 10.1016/j.isci.2023.106993

**Published:** 2023-05-29

**Authors:** Eleonora Lomi, Kate J. Jeffery, Anna S. Mitchell

**Affiliations:** 1 Department of Experimental Psychology, University of Oxford, The Tinsley Building, Mansfield Road, OX1 3SR Oxford, UK; 2 School of Psychology & Neuroscience, College of Medical, Veterinary & Life Sciences, University of Glasgow, G12 8QB Glasgow, UK

**Keywords:** Behavioral neuroscience, Cognitive neuroscience, Neuroscience

## Abstract

The thalamus and cortex are anatomically interconnected, with the thalamus providing integral information for cortical functions. The anteroventral thalamic nucleus (AV) is reciprocally connected to retrosplenial cortex (RSC). Two distinct AV subfields, dorsomedial (AVDM) and ventrolateral (AVVL), project differentially to granular vs. dysgranular RSC, respectively. To probe if functional responses of AV neurons differ, we recorded single neurons and local field potentials from AVDM and AVVL in rats during foraging. We observed place cells (neurons modulated by spatial location) in both AVDM and AVVL. Additionally, we characterized neurons modulated by theta oscillations, heading direction, and a conjunction of these. Place cells and conjunctive Theta-by-Head direction cells were more prevalent in AVVL; more non-conjunctive theta and directional neurons were prevalent in AVDM. These findings add further evidence that there are two thalamocortical circuits connecting AV and RSC, and reveal that the signaling involves place information in addition to direction and theta.

## Highlights

• Anteroventral thalamic (AV) neurons are modulated by spatial location
• AV place cells have different firing field metrics to hippocampal place cells
• AV neurons are also modulated by theta, head direction, and self-motion cues
• Two AV directional signals: broad at theta frequency vs. narrow theta independent

## Graphical abstract

**Figure:**
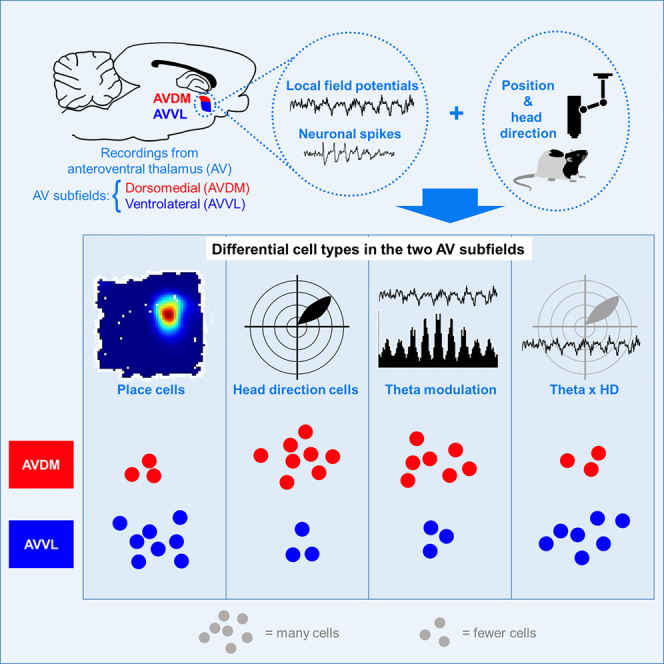

## Introduction

A key objective in neuroscience is to identify the neural circuitry underlying the mammalian sense of direction, which provides the foundation for spatial representations and navigation, as well as episodic memory functions.^1^^,^^2^^,^^3^^,^^4^^,^^5^^,^^6^^,^^7^ The directional representation is supported by head direction (HD) cells, which are active when the animal faces a cell’s preferred firing direction (PFD).^8^ In rodents, HD cells have been recorded within interdependent neural networks of cortical and subcortical structures including anterior thalamus, lateral mammillary bodies, and retrosplenial cortex (RSC). This system of interconnected structures has been referred to as a “tripartite system” for spatial and memory functions,^9^ superseding the notion of an “extended hippocampal system”.^10^^,^^11^

HD neurons in RSC form two subpopulations, one in the dysgranular RSC subregion (dRSC) that is more influenced by visual landmarks and egocentric sensory signals,^12^^,^^13^ and one distributed across both dRSC and granular (gRSC) subregions, comprising the “classic” HD cell population that computes global heading direction of the animal.^14^ To understand how RSC HD cells construct their signals, it is necessary to determine what information is carried by their thalamocortical inputs. The RSC is reciprocally connected with the anterodorsal (AD) and anteroventral (AV) thalamic nuclei of the anterior thalamus.^15^^,^^16^^,^^17^^,^^18^^,^^19^^,^^20^ Past work has focused on the AD as the main subcortical source of HD information to cortex.^7^^,^^21^^,^^22^^,^^23^ While the majority of neurons in the AD are HD cells, the general consensus for the AV is that cells here are less directionally modulated but more strongly modulated by theta frequency oscillations.^24^^,^^25^^,^^26^^,^^27^^,^^28^^,^^29^ Most AV cells discharge rhythmically and coherently with local and hippocampal theta oscillations.^27^ However, evidence has also implicated the AV in directional processing, as HD cells have been reported here.^26^ In the most dorsomedial subfield of AV (AVDM), at the border with AD, 39% of these units were conjunctively theta- and HD-modulated,^26^ a property not observed along the main HD circuit via the AD.^1^^,^^22^ This suggests that AD and AV neural signals passed between the RSC and other interconnected cortical network structures can be functionally differentiated.

**Figure 1:**
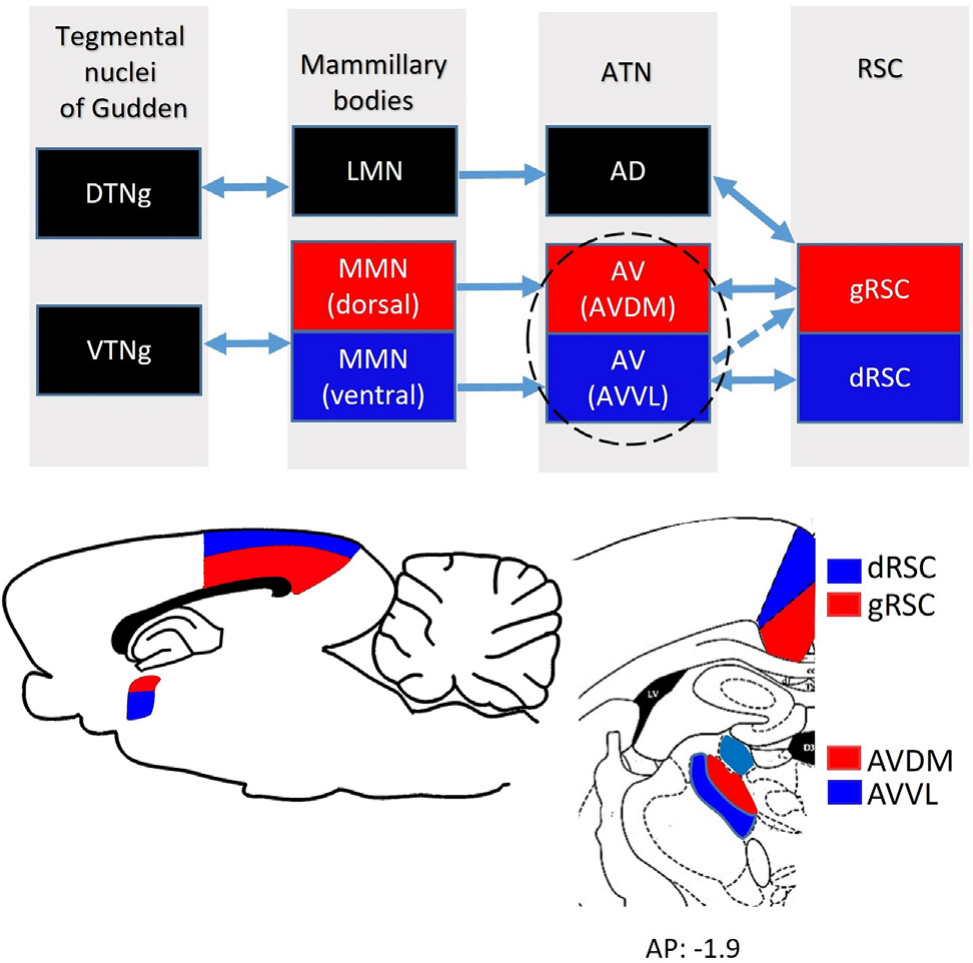
The distinct pathways for thalamocortical interactions with retrosplenial cortex (RSC) head direction functioning The tegmental nuclei of Gudden in the brainstem comprise separate dorsal (DTNg) and ventral (VTNg) tegmental nuclei that project to the lateral (LMN) and the medial (MMN) nuclei of the mammillary bodies, respectively. LMN projects to anterodorsal (AD) nucleus of the anterior thalamic nuclei (ATN). MMN has separate dorsal (red) and ventral (blue) pathways that project to the RSC via the anteroventral thalamic nucleus (AV). Dorsal MMN projects to dorsomedial AV subfield (AVDM); ventral MMN projects to ventrolateral AV subfield (AVVL). In turn, AVDM projects to granular RSC (gRSC) and AVVL projects strongly to dysgranular RSC (dRSC) and less to gRSC (represented by a dashed line to indicate differences in projection strength). AVDM and AVVL (encircled) are the sites of our recordings. Bottom left, Sagittal schematic diagram showing the anatomical location of dRSC (blue) and gRSC (red) subregions, AD (red) and AV (blue). Bottom right, coronal rat brain atlas section showing dRSC, gRSC and AVDM and AVVL subfields, adapted from Paxinos and Watson.^37^ AD is marked in light blue. AP, anterior-posterior level of the section from Bregma (mm).

There also seems to be functional differentiation *within* AV. Recent work, including ours, has shown that AV-RSC projections comprise distinct pathways.^16^^,^^17^^,^^30^^,^^31^^,^^32^^,^^33^ One thalamocortical pathway projects from AVDM to gRSC (Figure 1). This gRSC subregion also receives projections from the hippocampus via the subiculum.^31^^,^^32^^,^^33^^,^^34^^,^^35^ The other pathway projects from the ventrolateral AV subfield (AVVL) to dRSC. This dRSC subregion is more closely linked to visual structures.^32^^,^^36^

The notion of different AV neuronal responses is further reinforced by examination of the inputs to AV (Figure 1). The main input to the two AV subfields arrives from anatomically segregated populations of cells in the medial nuclei of the mammillary bodies (MMN).^19^^,^^34^^,^^38^ Specifically, dorsal MMN projects to AVDM, while ventral MMN projects to AVVL. In turn, MMN receives inputs from the medial septum^39^^,^^40^ and ventral tegmental nucleus of Gudden (VTNg),^34^ providing a source of theta information to AV.^41^^,^^42^^,^^43^ Additionally, AV receives hippocampal information directly via the dorsal subiculum.^5^^,^^17^^,^^18^^,^^32^^,^^38^^,^^44^

Given this distinct neuroanatomical connectivity of the AVDM and AVVL, we recorded single neuron spiking and local field potentials (LFP) using tetrodes within these two AV subfields in foraging rats to understand what information is carried by AV inputs to the cortical HD cells. We found that a notable percentage of cells were place cells, showing spatially localized firing. While place cells were found throughout both AV subfields, there were a higher number recorded in AVVL. AV place cells showed some differences from classic hippocampal place cells, as well as many similarities including phase precession. Additionally, both AV subfields contained functionally distinct populations of neurons that were modulated by several spatial signals including HD and self-motion (including theta and head movement speed). When we compared between AVDM and AVVL, we did not find any differences in overall directionality, nor in theta modulation. However, there were fewer conjunctive cells in AVDM, and correspondingly more that were only directional or only theta modulated. Overall, our findings extend observations that the two AV subfields have anatomically and functionally distinct subdivisions that signal and integrate place information, in addition to direction and theta.

## Results

### Histology

**Figure 2:**
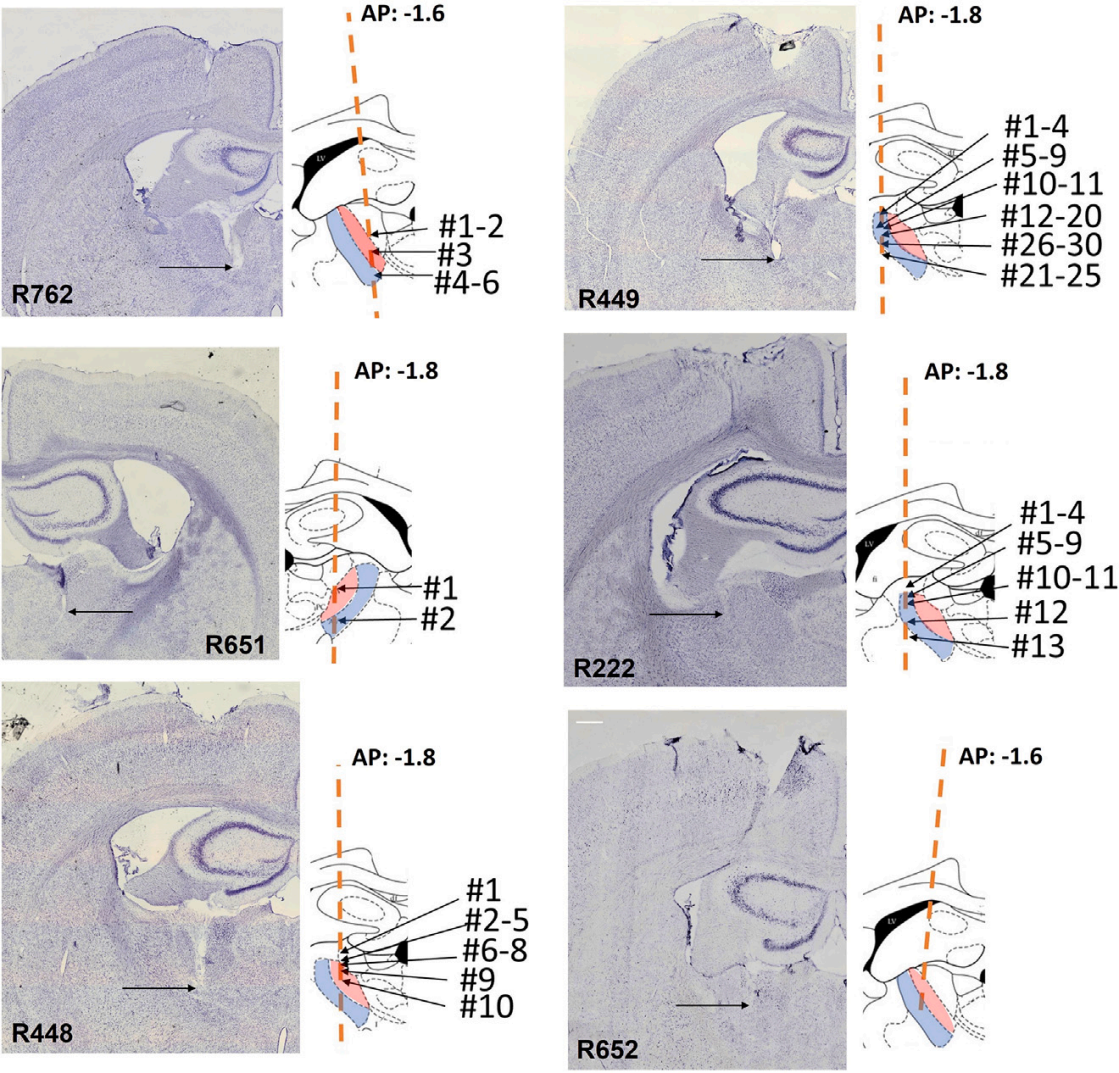
Histology of anteroventral thalamic nucleus (AV) single-unit recordings Photomicrographs of the Nissl-stained coronal sections through the recording site, for the six implanted rats. Insets on the right show schematic line drawing of AV subfields (AVDM, red; AVVL, blue) for each animal**,** adapted from Paxinos and Watson.^37^ Locations of place cells are marked for the five rats in which place cells could be recorded. Place cell location is reconstructed on the atlas section and is indicated by numbers. Orange line shows the estimated track direction based on histology as all tetrodes were implanted with no angle. Anterior-posterior (AP) location of each atlas section is indicated (−1.6 or −1.8mm from Bregma). Abbreviations: AVDM, dorsomedial anteroventral thalamic nucleus; AVVL, ventrolateral anteroventral thalamic nucleus. Scale bar: 200μm.

Cell locations were estimated based on the reconstruction of the electrode tracks from histology and the experiment records documenting screw turns that lowered the electrodes each day, starting from a known depth. Figure 2 illustrates examples of brain sections in which the electrode track is visible. The electrode’s tip is marked and represents the deepest recording site. Two rats (R449, R222) had AVVL-only recordings; one (R762) had AVDM-only recordings. The remaining three (R448, R651 and R652) had both AVDM and AVVL recordings. See details on recording sites in Table 2. To be sure that the place cells were indeed located in AV, in two animals (R448 and R449) we induced radio-frequency current lesions at the end of recording, through electrodes that had recorded both a Theta-by-HD and a place cell on that day. The lesion verified that the tip of the electrodes was located in AV; no damage to hippocampal and parahippocampal tissue was visible in the histological slides that contained these tracks, and there was no evidence of any pulling-down of hippocampal tissue into the anterior thalamic region. We are thus confident that these were thalamic neurons.

### Multiple functional cell types in AV

**Figure 3:**
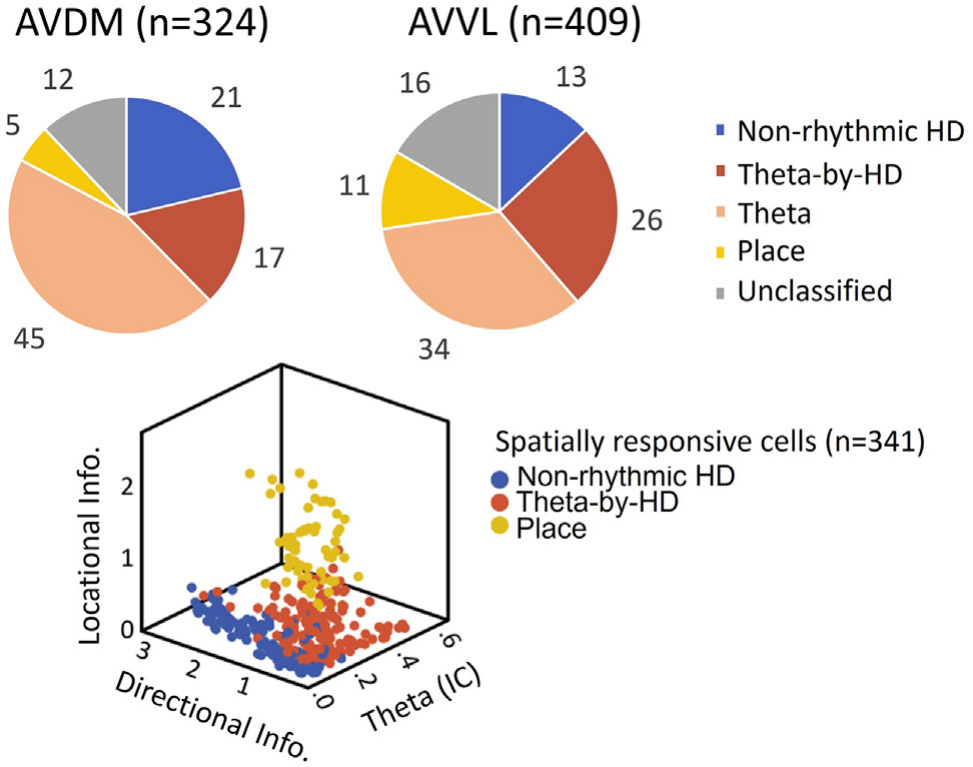
Different anatomical distributions of functional cell types within two anteroventral thalamic nucleus (AV) subfields Top, proportion of cells in the two AV subfields: dorsomedial (AVDM) and ventrolateral (AVVL). Cell numbers are as follows (AVDM vs. AVVL): non-rhythmic head direction (HD) (122), 69 vs. 53; Theta-by-HD, 53 vs. 105; place, 17 vs. 44; Theta, 146 vs. 139; Unidentified, 39 vs. 68. ∗∗ = 0.01 significance, Chi-squared test. There was no difference in the anatomical location of the unclassified units with no spatial/temporal correlates. Bottom, the three groups of spatial/directional cells were well segregated in a scatterplot along three independent parameters.

For the analysis, we used the six rats for which histology indicated that electrodes sampled the AV (see Table 1). A total of 733 cells were collected from the six animals moving freely in the open field box (n = 324 in AVDM, n = 409 in AVVL; see Figure 3 and Tables 1 and 2 for breakdown of cell types and AV subfields). Of these, 504 (69%) cells were significantly theta-modulated, 280 (38%) were directionally modulated, and 61 (8%) were place cells. All place cells were theta-modulated while, among the directional neurons, 158 (22% of all recorded units; 56.43% of the directional units) were conjunctive for theta and head direction modulation. The remaining 107 cells did not fit into any clear classification.

**Table 1:** Summary of recording locations and cell distribution in anteroventral thalamic nucleus (AV) for all animals (n = 733 cells in six animals)

Recorded cells			
Rat	Hemisphere	Region	N
R222	Left	AVDM AVVL	0 24
R448	Left	AVDM AVVL	106 76
R449	Left	AVDM AVVL	0 211
R651	Right	AVDM AVVL	31 66
R652	Left	AVDM AVVL	116 32
R762	Left	AVDM AVVL	71 0
**All (n = 6)**		**AVDM** **AVVL**	**324** **409**

AVDM = dorsomedial subfield of the AV; AVVL = ventrolateral subfield of the AV.

**Table 2:** Numbers and percentages of anteroventral thalamic nucleus (AV) cell types recorded in each animal

Rat	N	Non-rhythmic HD	Theta-by-HD	Place	Theta	Unclassified
R222	24	0	3 (13%)	13 (54%)	8 (33%)	0
R448	182	30 (17%)	31 (17%)	10 (6%)	88 (48%)	23 (13%)
R449	211	2 (1%)	53 (25%)	30 (14%)	81 (38%)	45 (21%)
R651	97	3 (3%)	37 (38%)	2 (2%)	34 (35%)	21 (22%)
R652	148	80 (54%)	19 (13%)	0	46 (31%)	3 (2%)
R762	71	7 (10%)	15 (24%)	6 (9%)	28 (39%)	15 (21%)
**All**	**733**	**122 (17%)**	**158 (22%)**	**61 (8%)**	**285 (39%)**	**107 (15%)**

HD = head direction cells.

When we compared AVDM and AVVL subfields we did not find any difference in overall directionality (38% and 39%, respectively) or in theta modulation (61% and 60%, respectively). However, there were fewer conjunctive cells in AVDM (16% vs. 26%; χ2 = 9.28, p = 0.0023) and correspondingly more that were only directional (21% vs. 13%; χ2 = 9.06, p = 0.0029) or only theta-modulated (45% vs. 34%; χ2 = 9.33, p = 0.0022). These cell types (Figure 3) and their properties are examined in turn, below.

We start with the spatial/directional cells (examples in Figures S1–S4). As expected, based on previous work, HD cells (n = 280; 38%) were found throughout the AV subfields: AVDM = 122; 44%; AVVL = 158; 65%. These consisted of two subtypes, with distinct directional properties (analysis reported below).

**Figure 4:**
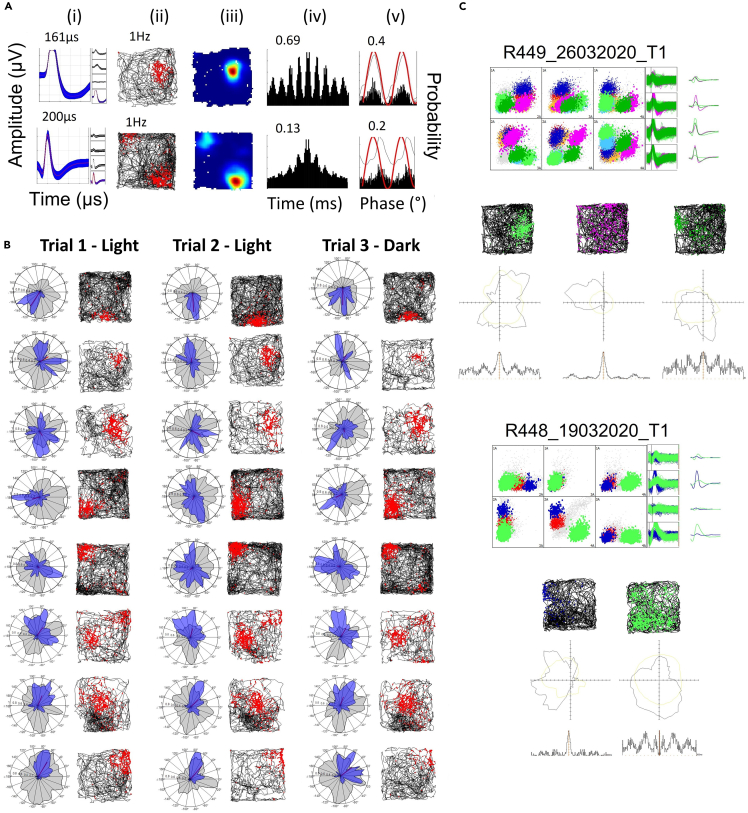
Place cells in the anteroventral thalamic nucleus are highly stable across trials and can be co-recorded on the same tetrode as directional neurons (A) Examples of place cells with single and multiple firing fields. For each cell the following plots are shown: (i) Average tetrode spike waveform. Red line is the mean, shaded area the standard deviation. Average peak-to-trough width (μs) values are reported on top. (ii) Spikemap and (iii) ratemap with mean firing rate (Hz), showing uniform distribution of spikes across the box. (iv) Temporal autocorrelogram implemented between ±500ms with index of rhythmicity (IR). (v) Double-plotted histogram of spike frequency relative to theta phases. The thin line is the corresponding smoothed density estimates (see STAR Methods). The red line is the sine wave representing two theta cycles. x axis, degrees between 0 and 360; y axis, probability. (B) Stability of place fields across trials and in darkness. Spikemaps and polar plots of firing rate vs. 360° of HD (blue) and dwelltime vs. HD (gray) are shown for each trial (each column is a trial; third is in darkness). Ratemap correlations were 0.9 ± 0.01 in Light-Light and 0.87 ± 0.03 in Light-Dark trials. (C) Examples of two recording trials in which place cells were co-recorded on the same tetrode as a Theta-by-HD cell. The tetrode cluster space and the average tetrode waveform for each cluster are shown. Spikemap, polar plot of firing rate (Hz) vs. HD (degrees) and temporal autocorrelogram between ±300ms are shown for each unit.

The most notable observation was that a subset of cells from 5/6 animals (n = 61; 8.32%) were place cells (Figure 4A), which have not been reported previously in AV. These cells were recorded throughout both AV subfields (AVDM = 17; 5%; AVVL = 44; 11%) and formed single (Figure S3) or multiple (Figure S4) discrete firing fields. Fields were always highly compact and stable across trials. In two animals, we recorded seven place cells simultaneously on the same tetrode as one or two Theta-by-HD cells, across six different days (see examples in Figure 4C), confirming that recorded place cells were part of the local AV circuit and not ectopic cells dragged down from the overlying hippocampus (which anyway would have been unlikely, given the distance).

Having provided an overview of the functional cell types recorded, we next took each of these cell types in turn and investigated their physiological properties and firing correlates (summarized in Table 3).

**Table 3:** Electrophysiological properties of functional cell types in anteroventral thalamic nucleus (AV) by subfield

	Non-rhythmic HD	Theta-by-HD	Theta non-dir.	Place
N cells				
Total (/733)	122 (17%)	158 (22%)	285 (39%)	61 (8%)
AVDM (/324)	69 (21%)	53 (17%)	146 (45%)	17 (5%)
AVVL (/409)	53 (13%)	105 (26%)	139 (34%)	44 (11%)
Spike width (μs)				
Total	258.36 ± 13.73	267.98 ± 10.39	344.77 ± 9.70	284.26 ± 20.35
AVDM	258.26 ± 14.84	320.38 ± 19.1	366.03 ± 14.91	190.59 ± 16.81
AVVL	358.49 ± 25.21	241.52 ± 11.54	322.45 ± 12.03	320.45 ± 25.51
Mean rate (Hz)				
Total	4.64 ± 0.42	2.55 ± 0.18	4.27 ± 0.32	1.1 ± 0.1
AVDM	3.45 ± 0.24	2.42 ± 0.29	4.70 ± 0.52	0.83 ± 0.17
AVVL	6.19 ± 0.74	2.62 ± 0.23	3.85 ± 0.38	1.20 ± 0.12
Peak rate (Hz)				
Total	27.06 ± 2.92	5.20 ± 0.31	5.57 ± 0.38	11.29 ± 0.96
AVDM	19.57 ± 3.16	5.23 ± 0.36	6.07 ± 0.61	9.42 ± 1.86
AVVL	36.80 ± 5.04	5.16 ± 0.60	5.06 ± 0.44	12.01 ± 1.11
Index of coupling (IC)				
Total	0.05 ± 0.00	0.17 ± 0.01	0.23 ± 0.01	0.15 ± 0.01
AVDM	0.05 ± 0.00	0.19 ± 0.02	0.25 ± 0.01	0.17 ± 0.02
AVVL	0.05 ± 0.01	0.16 ± 0.01	0.20 ± 0.01	0.14 ± 0.01
Index of rhythmicity (IR)				
Total	−0.004 ± 0.01	0.26 ± 0.01	0.24 ± 0.01	0.24 ± 0.02
AVDM	0.007 ± 0.02	0.26 ± 0.03	0.26 ± 0.02	0.24 ± 0.04
AVVL	−0.02 ± 0.02	0.26 ± 0.02	0.20 ± 0.01	0.24 ± 0.03
Bursting cell proportion				
Total	9/122 (7%)	74/158 (47%)	95/285 (33%)	42/61 (69%)
AVDM	5/69 (7%)	24/53 (45%)	46/146 (34%)	13/17 (76%)
AVVL	4/53 (8%)	50/105 (48%)	49/139 (35%)	29/44 (66%)
Peak ISI (ms)				
Total	23.89 ± 3.16	10.24 ± 1.19	18.29 ± 1.89	5.75 ± 0.22
AVDM	31.72 ± 5.34	12.73 ± 2.88	18.67 ± 2.71	5.47 ± 0.32
AVVL	13.68 ± 1.11	8.98 ± 1.05	17.89 ± 2.64	5.86 ± 0.27
Directional information (bits/spike)				
Total	1.10 ± 0.06	0.32 ± 0.02	0.05 ± 0.00	0.40 ± 0.04
AVDM	0.96 ± 0.08	0.34 ± 0.05	0.05 ± 0.00	0.58 ± 0.1
AVVL	1.28 ± 0.10	0.31 ± 0.02	0.05 ± 0.00	0.33 ± 0.04
Spatial information. (bits/spike)				
Total	0.08 ± 0.01	0.31 ± 0.02	0.12 ± 0.01	1.37 ± 0.06
AVDM	0.09 ± 0.02	0.31 ± 0.04	0.10 ± 0.01	1.36 ± 0.11
AVVL	0.07 ± 0.01	0.31 ± 0.02	0.13 ± 0.02	1.38 ± 0.07
Sparsity score				
Total	0.56 ± 0.01	0.49 ± 0.01	0.65 ± 0.01	0.15 ± 0.01
AVDM	0.55 ± 0.01	0.48 ± 0.02	0.65 ± 0.01	0.13 ± 0.01
AVVL	0.56 ± 0.01	0.49 ± 0.02	0.66 ± 0.01	0.16 ± 0.01
Speed cell proportion				
Total	98/122 (80%)	121/158 (77%)	200/285 (70%)	50/61 (82%)
AVDM	55/69 (80%)	38/53 (72%)	99/146 (68%)	12/17 (71%)
AVVL	43/53 (81%)	83/105 (79%)	101/139 (73%)	38/44 (87%)
Angular head velocity (AHV) cell proportion				
Total	11/122 (9%)	24/158 (15%)	0/285	9/61 (15%)
AVDM	7/69 (10%)	12/53 (23%)	0/146	1/17(6%)
AVVL	4/53 (8%)	12/105 (11%)	0/139	8/44 (18%)

AVDM = dorsomedial subfield of the AV; AVVL = ventrolateral subfield of the AV; HD = head direction cells.

### Spatial firing

Cells were examined for the presence or absence of spatial firing patterns. Sixty-one cells (8%) were found to have place fields. These AV place cells primarily formed single place fields in the 90 × 90cm box (n = 5/61 had 2-3 fields regularly spaced), like hippocampal neurons recorded in similar-sized environments.^45^^,^^46^ Place cells were characterized by high locational information (1.51 ± 0.06 bits/spike) and locational selectivity (15.15 ± 1.13), and low sparsity (0.15 ± 0.01) compared to HD cells (locational information: 0.35 ± 0.02 bits/spike; selectivity: 4.24 ± 0.14; sparsity: 0.52 ± 0.9; Table 3).

We looked at whether there were AV subfield differences in the cells’ spatial properties. We found no difference in place field size between AVDM (655.76 ± 76.50 cm^2^) and AVVL (741.59 ± 54.68 cm^2^; two-sample t-test, *t*(59) = −0.86, SD = 87.62, p = 0.39). However, consistent with the larger concentration of place cells in AVVL, shown above, spatial modulation was higher in AVVL (locational information: 0.26 ± 0.02 bits/spike) compared to AVDM (0.26 ± 0.02 bits/spike; two-sample WRS, z = −3.32, p < 0.001). Directional modulation was the same in both (AVDM: 0.33 ± 0.03 bits/spike; R-vector: 0.25 ± 0.02; AVVL: 0.32 ± 0.02 bits/spike; z = −0.45, p = 0.653; R-vector: 0.26 ± 0.01; z = −1.45, p = 0.146).

While testing in the dark did not cause place remapping (Figure S8), nor a change in global mean firing rate (z = −0.96, p > 0.1), locational information was reduced in dark compared to light conditions (paired-sample WSR test, z = 4.16, p < 0.0001). It is possible that a slight place field drift occurred in the absence of visual anchoring. Ratemap correlations were 0.9 ± 0.01 in light-light and 0.87 ± 0.03 in light-dark. There was no difference between conditions (two-sample KS test; ks-stat = 0.14, p = 0.74). Observed and control distribution of correlation coefficients differed significantly from each other (two-sample KS test, Light-Light, ks-stat = 0.86; light-dark, ks-stat = 0.853; all p < 0.0001).

### Spatial firing differs from hippocampus

We compared the spatial properties and field metrics of AV place cells with hippocampal CA1 place cells (n = 55) previously recorded in a similar open field (Casali et al.^47^; see STAR Methods). In the AV data, place fields were smaller and more spatially compact (mean field area: 717.67 ± 44.78 cm^2^. Sparsity: 0.15 ± 0.01) compared to CA1 place cells (mean field area: 1236.50 ± 154.20 cm^2^; *t*(114) = −3.37, SD = 206.76, p = 0.001. Sparsity: 0.18 ± 0.01; *t*(114) = −4.08, SD = 0.09, p = 0.017). Spatial information was lower in AV compared to CA1 (1.37 ± 0.06 vs. 1.75 ± 0.13; *t*(114) = −2.81, SD = 0.73, p = 0.006). Directional information was the same for both AV and CA1 (0.40 ± 0.01 vs. 0.29 ± 0.05; *t*(114) = 1.73, SD = 0.34, p = 0.09), supporting the notion that AV place cells were purely spatial.

In terms of temporal firing properties, average waveform width was the same for AV and CA1 place cells (284.26 ± 20.35 vs. 304.73 ± 15.09; *t*(114) = −0.78, SD = 138.64, p = 0.43), reinforcing the conclusion that AV place cells did not result from axon spikes. AV place cells fired with higher mean and peak rates compared to CA1 (mean: 1.1 ± 0.1 vs. 0.71 ± 0.08; 0.08; *t*(114) = 2.96, SD = 0.7, p = 0.004. Peak: 11.3 ± 0.96 vs. 7.23 ± 0.53; *t*(114) = 3.6, SD = 6.06, p < 0.001) and showed stronger complex spike bursting (burst index: 0.18 ± 0.01 vs. 0.14 ± 0.01; *t*(114) = 2.09, SD = 0.08, p = 0.04).

When we compared theta oscillatory firing, AV place cells were less theta-modulated than CA1 place cells (IC: 0.15 ± 0.01 vs. 0.22 ± 0.02, IR: 0.24 ± 0.02 vs. 0.37 ± 0.04; all p < 0.01). However, a higher number of AV place cell showed phase precession (77.05% vs. 25.5%; χ^2^ = 30.88, p < 0.0001), consistent with previous observations that phase precession is weak for hippocampal place cells recorded in the open field.^48^

Finally, in terms of velocity sensitivity, the same proportions of AV and CA1 place cells were speed modulated (82% vs. 73%; χ^2^ = 1.42, p = 0.23), while only AV place cells showed additional angular head velocity (AHV) modulation (15% vs. 0; χ^2^ = 8.79, p = 0.003). These firing properties will be explored in turn for all AV cell groups next.

### Two types of directional firing

**Figure 5:**
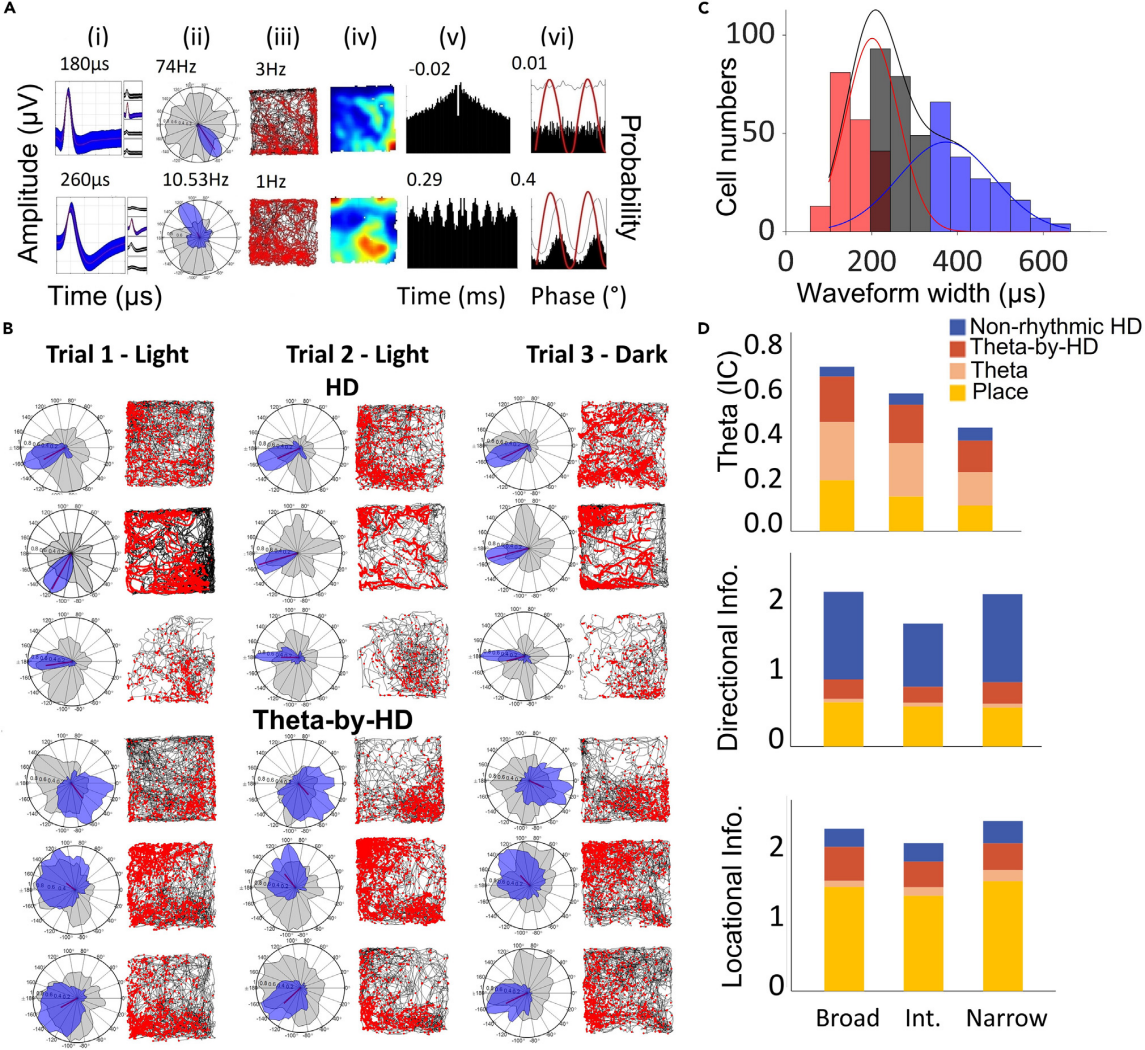
Two types of directional cells in the anteroventral thalamic nucleus (AV): theta-rhythmic and non-rhythmic (A) An example of each is presented. Plots as in Figure 4A. In the polar plots, firing rate (Hz) vs. head direction (degrees) is shown in blue and dwelltime (seconds) vs. head direction (HD; degrees) is shown in gray. Directional peak firing rates are reported. (B) Examples of HD and Theta-by-HD recorded across three consecutive trials. Plot as in Figure 4B. (C) Frequency distribution of peak-to-trough waveform width for AV cells. x axis, μs; y axis, frequency count. Gaussian fits for the different modes (waveform types) are displayed: broad (blue), narrow (red), and intermediate (gray). See also Figure S5.(D) For each cell type, distribution on theta, directional and locational properties are presented as stacked bar charts, with cell types as grouping variable. Theta is more likely to occur in broad compared to narrow and intermediate waveforms. In contrast, directionality is more likely to occur in narrow and intermediate compared to broad waveforms. Locational selectivity is equally represented.

Having examined the spatial firing characteristics of AV units, we now move to their directional properties (Figures 5A and 5B). 288 (38%) cells had significant directionality in their firing. As mentioned earlier, both theta-modulated (Theta-by-HD) and non-theta-modulated (HD) cells were found in both AV subfields, but the non-rhythmic HD cells were more prevalent in AVDM (χ2 = 9.06, p = 0.003) and the rhythmic HD cells more prevalent in AVVL (χ2 = 9.28, p = 0.002).

**Figure 6:**
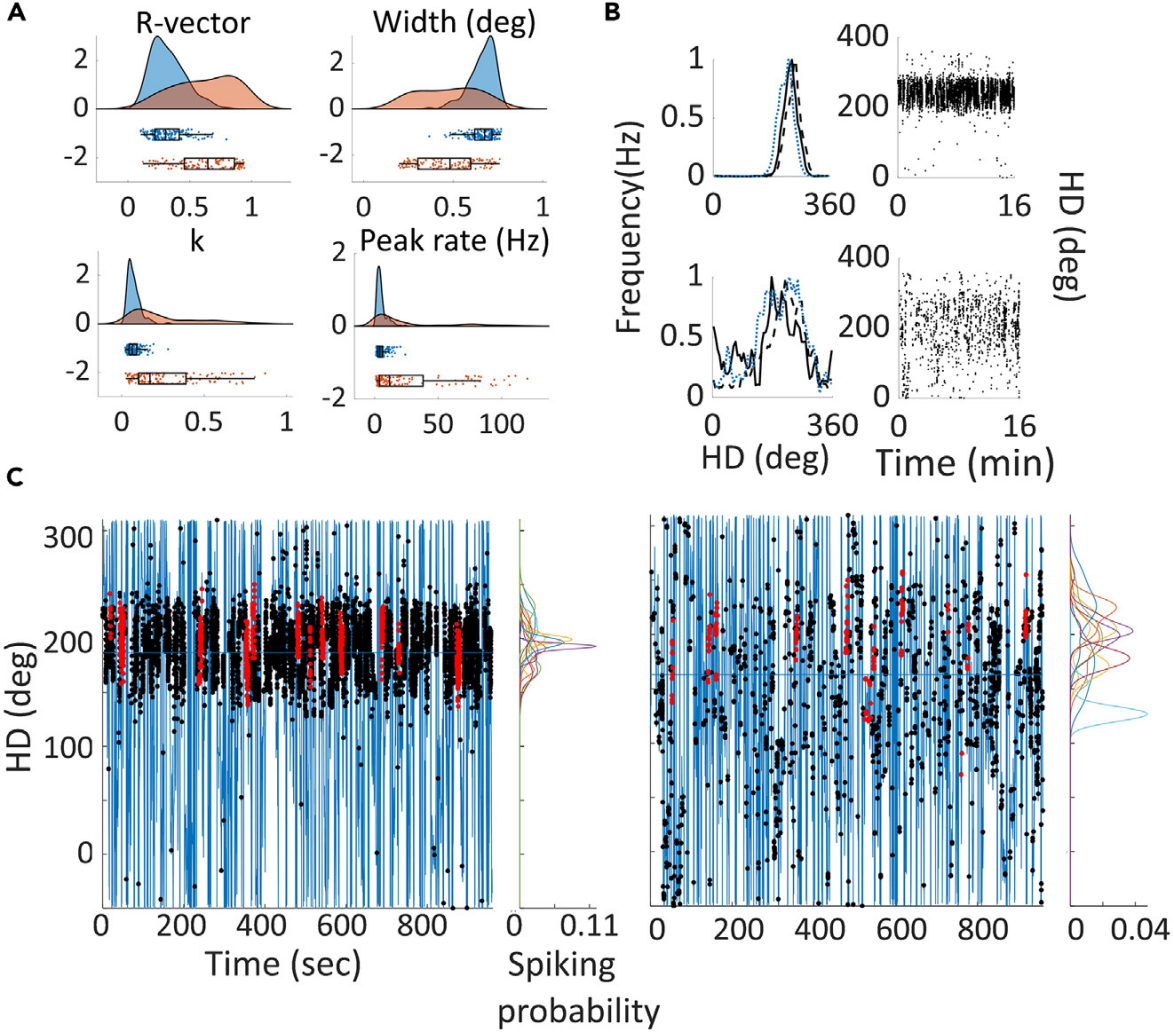
Theta-by-Head direction (HD) cells show lower directional specificity than non-theta HD cells (A) Distribution of tuning curve parameters, shown as raw data + boxplot + split-violin plot. k, concentration parameter. (B) Peak normalized tuning curves for non-theta HD (top) and Theta-by-HD (bottom) cells, calculated over short timescales: solid line, first 3 min; dashed line, last 3 min; blue line, remaining trial duration. Theta-by-HD cell tuning curves are broader from the start. For each cell, the corresponding scatterplot of HD vs. time are shown. (C) For the same cells in B, spatial plots of unsmoothed directional heading as a function of time are plotted (blue line), overlaid at the appropriate times with spikes (black markers). Red markers are spikes emitted during full head turn events through the cells’ preferred firing direction (when the cells fired >50% of its intra-trial peak rate plus spikes within +/−50°). Kernel density estimates (KDE) for each successive event are plotted on the right and show a larger variability for Theta-by-HD cells.

There were other differences between the two cell types (Figure 6A). Theta-by-HD cells displayed less-precise directional firing compared to non-rhythmic HD cells: Theta-by-HD cells showed lower R-vectors (0.62 ± 0.02 vs. 0.33 ± 0.02; two-sample WRS test, z = −9.74), broader tuning widths (94.04 ± 3.0 vs. 132.30 ± 1.15; z = −9.74) and smaller peak rates (27.06 ± 2.92 vs. 5.20 ± 0.03Hz; z = 6.59). The concentration parameter, which provides a measure of how directionally compact the firing is (like the sparsity score used for place cells), was lower for Theta-by-HD cells (0.72 ± 0.03 vs. 2.53 ± 0.19; z = 9.74; all p < 0.001)*.*

To explore why Theta-by-HD cells had broader tuning curves, we looked at the tuning across a trial and found that Theta-by-HD cells shifted directions more during a trial, which could have led to a broader tuning curve when collapsed across the trial (Figure 6C). Specifically, Theta-by-HD cells displayed larger jitter in the HD data when they fired more than 50% of intra-trial peak rate (SEM of peak location: non-rhythmic HD, 13.66 ± 0.60°; Theta-by-HD, 16.59 ± 0.67°; two-sample *t*-test, t(244) = −3.16, SD = 7.21, p = 0.002). In contrast, KDE peaks for Theta-by-HD and non-rhythmic HD cells had an equivalent mean width, suggesting that the range of directional firing was actually the same (non-rhythmic HD, 33.9 ± 0.75; Theta-by-HD, 35.59 ± 0.68; t(252) = −1.67, SD = 8.02, p = 0.1). SEM of peak width was also the same (non-rhythmic HD, 4.52 ± 0.22; Theta-by-HD, 5.22 ± 0.28; t(244) = −1.92, SD = 2.87, p = 0.06).

However, not all the tuning curve broadening in Theta-by-HD cells could be explained by drift because at small time intervals, when drifting is small, we saw residual broadening. This was investigated by constructing plots of firing rate vs. HD for the first 3 min of recordings (Figure 6B). Average tuning width continued to be broader for Theta-by-HD (133.09 ± 1.33°) compared to non-rhythmic HD cells (97.43 ± 2.80°; two-sample WRS test, z = −9.56, p < 0.0001). Likewise, tuning curves remained broader when the animal was turning clockwise (CW), counter-clockwise (CCW) or when still (see supplemental information; Table 5), suggesting that a larger variability of heading directions might also be a fundamental property of these cells.

**Figure 7:**
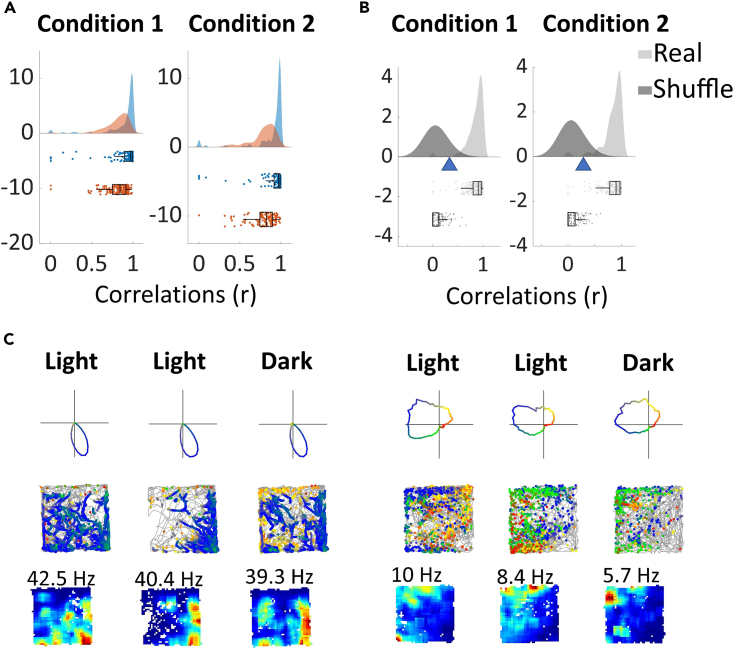
Directional firing is similar across light-light (Condition 1) and light-dark (Condition 2) trials For each Condition: (A) Distribution of trial pair-specific tuning curve correlations for non-rhythmic head direction (HD) (blue) and Theta-by-HD (red) cells. Ratemap correlations were as follows. light-light: non-rhythmic HD, 0.89 ± 0.19; Theta-by-HD, 0.81 ± 0.01. light-dark: non-rhythmic HD, 0.89 ± 0.02; Theta-by-HD, 0.79 ± 0.02. (B) Distribution of correlation coefficients for cells in A, compared to shuffle distributions, blue triangles represent the 95^th^ percentile shuffling. No difference between Conditions. (C) Examples of non-theta HD (left) and Theta-by-HD (right) cells recorded across the three trials. Each trial shows the following: Top, directionally color-coded polar plot, with direction of peak firing in blue, and the other directions following a color gradient changing every 90°; Middle, directionally color-coded spikemap, with spikes colored according to their direction overlaid on the path of the rat (highly smoothed in 20ms bins); Bottom, ratemap with peak rate reported. See Figure S8 for place cell examples.

Cells maintained stable PFDs across trials. Tuning curve correlations were high across both light-light trials (non-rhythmic HD, 0.89 ± 0.19; Theta-by-HD, 0.81 ± 0.01) and ight-dark trials (non-rhythmic HD, 0.89 ± 0.02; Theta-by-HD, 0.79 ± 0.02), with no difference between the two (two-sample KS test; non-rhythmic HD, ks-stat = 0.07; Theta-by-HD, ks-stat = 0.1; p > 0.5; Figure 7A). Observed and control distribution of correlation coefficients differed significantly from each other (Figure 7B) in both conditions for non-rhythmic HD cells (two-sample KS test, light-light, ks-stat = 0.94; light-dark, ks-stat = 0.94; all p < 0.0001) and Theta-by-HD cells (Light-Light, ks-stat = 0.92; light-dark, ks-stat = 0.95; all p < 0.0001). However, in line with the greater intra-trial drift for Theta-by-HD cells (shown above), correlations were higher for non-rhythmic HD cells across both light and dark conditions (two-sample KS test; light-light, ks-stat = 0.58; light-dark, ks-stat = 0.59; p < 0.0001). For both cell types, absence of vision did not affect coding of orientation (bits/spike; WSR test, non-rhythmic HD, z = −0.4631; Theta-by-HD, z = −0.23; p > 0.1) and overall firing rate (non-rhythmic HD, z = 1.66; Theta-by-HD, z = 2.40; p > 0.1 (Figure 7C).

### Place cells fields are not an artifact of directionality

**Figure 8:**
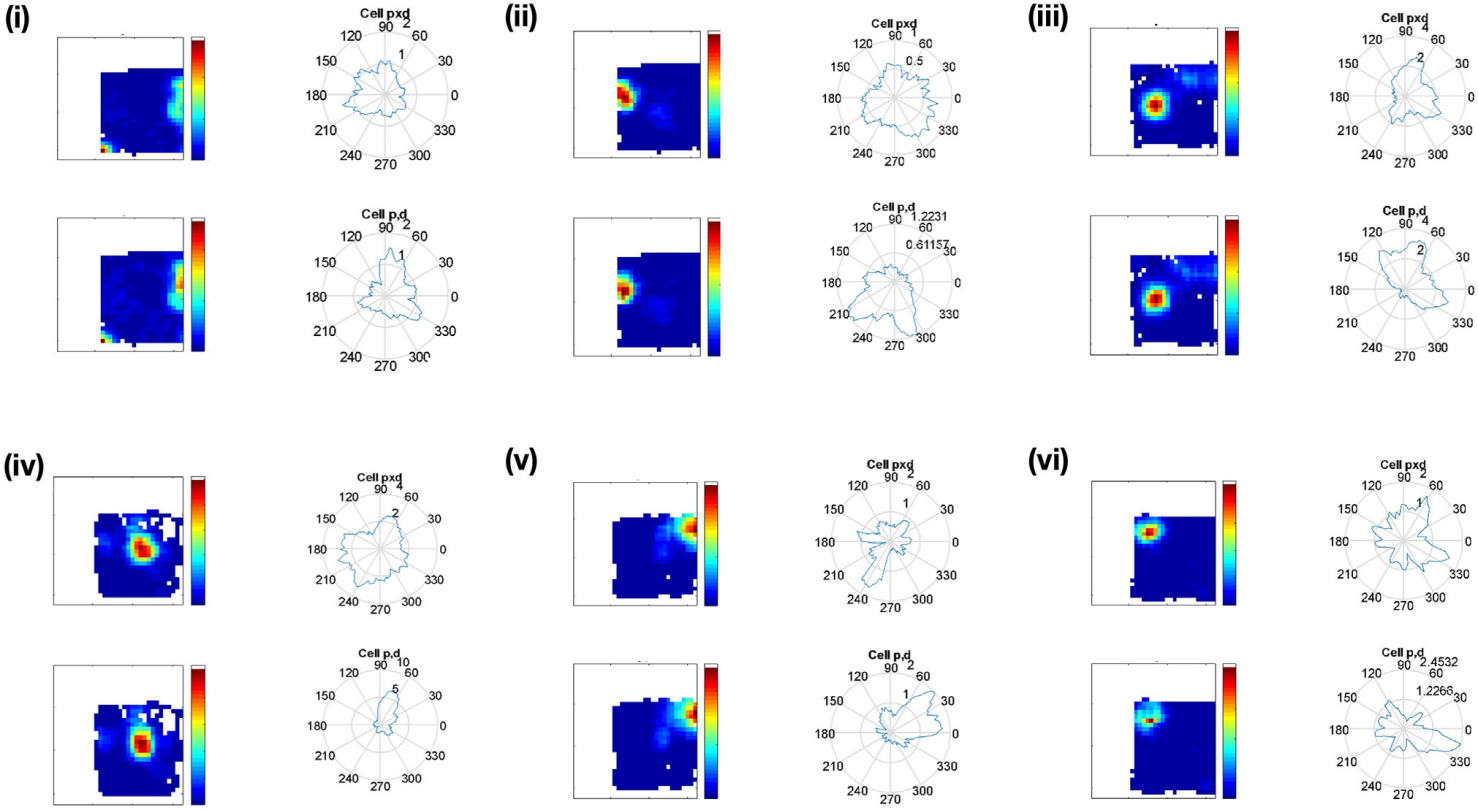
Place cell fields cannot be explained by the sampling bias The figure shows the effects of applying position-by-direction (pxd) corrections to the firing plots of place cells. Place fields cannot be explained by directionality as place fields are maintained after correcting for the sampling bias. For each cell, ratemaps and directional polar plots are shown before (“pd”) and after (“pxd”) isolating positional and directional spiking.

**Figure 9:**
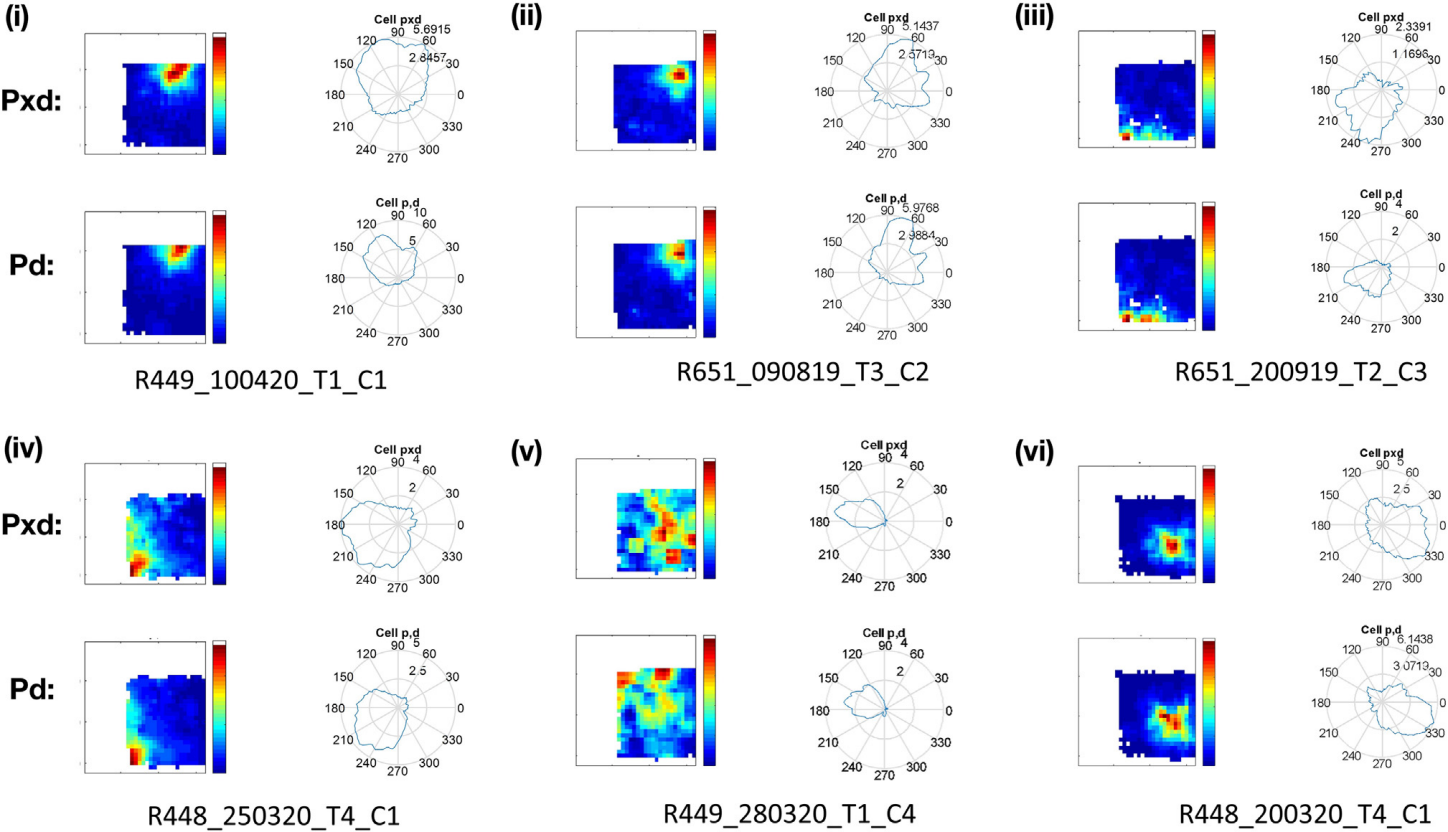
Theta-by-Head direction (HD) cells can sometimes show conjunctive spatial responses, not explained by the sampling bias The figure shows the effects of applying position-by-direction (pxd) corrections to the following Theta-by-HD cells, as per Figure 8. (i-iv) Same four cells as in the last four rows of Figure S2 (see Figure S2 caption). These cells are conjunctive, since both the preferred firing direction (PFD) and the place field are maintained after correcting for the sampling bias. (v) Theta-by-HD cell with a PFD in the polar plot, which should have developed a place field (according to the sampling bias hypothesis). However, the cell has no spatial selectivity, supporting the notion that spatial fields are not simply a by-product of directionality. (vi) Theta-by-HD cell with a place field away from boundaries, which would not be easily explainable by the sampling bias hypothesis i.e., the animal can approach this point from any direction.

Using the position-by-direction (pxd) analysis (see STAR Methods) to isolate directional and locational spiking (see STAR Methods), we investigated whether apparent firing properties such as location specificity might be artifacts, due to, for example, correlations between location and heading direction (Figure 8). Results of the pxd analysis are reported in Table 4, showing locational and directional information after isolating the two. Place cells remained purely locational (largely reduced directional information; Figure 8). By contrast, non-rhythmic HD cells were purely directional (largely reduced locational information). The effect of pxd analysis was more variable on Theta-by-HD cells (Figure 9; large range values). Among the directional units, 13% of the Theta-by-HD cells (n = 20/158) also passed the place cell criteria, although spatial tuning was much less specific than for place cells (Table 3) and the cells always fired over more than 20% of the arena. This is reflected in the ratemaps, which occasionally displayed broad patches of spatially inhomogeneous firing (see examples in Figure S2, last four rows).

**Table 4:** Percentage of reduction in locational and directional information in bits/spike after applying the position-by-direction (pxd) algorithm

% reduction in information content	Non-rhythmic HD	Theta-by-HD	Place cells
Directional information	−2.4% (−47%–21%)	−2.9% (−68%–57%)	−34.5% (−79%–9.4%)
Locational information	−68.2% (−96%–15%)	−23.2% (−84%–47%)	−7% (−25%–13%)

HD = head direction cells.

### Waveform analysis reveals different neuronal subtypes

We asked whether the cell groups identified in AV presented differences in their waveform shape, as this can be indicative of the AV cell typology.^49^^,^^50^ Approximately half of the spatial units (193/341, 56.6%) had a narrow peak-to-trough waveform (<250μs): non-rhythmic HD, 58.20%; Theta-by-HD, 53.80%; place cells, 56.60% (Table 3). Average width did not differ between these groups (KW test, H(3) = 49.54; p < 0.001; all p > 0.5) but all groups had a narrower width than the theta cells with no spatial/directional properties (all p < 0.01), for which only 32.28% of spikes were narrow.

We plotted the distribution of waveform widths (Figure 5C) and fitted a two-Gaussian model. Values fell into three distinct clusters, narrow (n = 192; 30.70%), broad (n = 183; 37.08%), and intermediate (n = 221; 32.22%). Cells were discarded if spike waveforms did not reach time for repolarization (the global minimum of the curve occurs before the peak, n = 30; 5.03%). The calibrated Hartigan’s dip test discarded the null hypothesis of unimodality (DIP = 0.04; p < 0.001). We compared the firing characteristics of cells in the three groups. The odds of finding a cell with a significant theta component in the broad spiking group was 5.8x (CI: 1.95, 6.56) and 3.21x (CI: 1.76, 5.85) higher, respectively, than in the narrow and intermediate groups, respectively (Fisher exact tests; all p < 0.0001). In contrast, cells with a significant HD component were 3.39x (CI: 2.201, 5.24) and 3.22x (CI: 2.11, 4.92) more likely to be found in the narrow and intermediate groups, respectively, than in the broad one (all p < 0.001). The odds of finding a cell with a place component were not significantly different for all three groups (all p > 0.05; Figure 5D). The variability of spike width (Figures S3 and S4) argues against place cells being axon spikes.

Peak-to-trough waveform width was greater for AVDM (330.24 ± 9.34μs) than AVVL neurons (284.83 ± 7.25μs; two-sample WRS, z = 4.26; p < 0.0001), as expected from their morphology, and may be a contributing factor for the variable waveform shapes reported above.

### Place cells show evidence of complex spike bursting

We next looked at the basic temporal patterns of firing (Figure S5). Place cells had the lowest mean rate, followed by Theta-by-HD cells and then non-rhythmic HD and theta non-directional cells, whose rate did not differ from each other (KW test; H(3) = 78.96; p < 0.001; HD vs. Theta-by-HD, p = 0.02; HD vs. theta, p = 1; HD vs. place, p < 0.001; place vs. Theta-by-HD, p < 0.01; place vs. theta, p < 0.01; Theta-by-HD vs. Theta, p < 0.01; Table 3). As expected from the waveform analysis above, there was a weak, but significant, negative correlation between peak-to-trough width and mean rate (Pearson’s r = −0.1; p = 0.01).

AV cells presented differences in a number of other temporal firing properties. Place cells contained the highest proportion of bursting units (69%), while Theta-by-HD (47%) and theta (33%) cells contained similar proportions of bursting and non-bursting units, and non-rhythmic HD cells only contained a marginal proportion of bursting units (7%). Proportions differed significantly from each other (Chi-square tests, all p < 0.001). This is visible as a high central peak for the autocorrelations of place cells (Figures S3 and S4), suggestive of complex spike bursting (higher than hippocampal CA1; shown above). Place and Theta-by-HD cells showed theta modulation, burst firing and a shorter ISI compared to non-rhythmic HD cells, which only fired single spikes that were mostly unrelated to theta (Table 3). Figure S5C shows examples of bursting and non-bursting units, and the distribution of peak ISI for the identified spatial cells.

Consistent with the larger concentration of place cells in AVVL (reported above), spike bursting propensity was higher in AVVL (peak ISI: 16.15 ± 1.28ms; burst index: 0.09 ± 0.00) compared to AVDM (peak ISI: 22.33 ± 2.00; two sample WRS, z = −3.49; p < 0.001; burst index: 0.08 ± 0.01; z = −2.87; p = 0.0041). There was no difference in mean firing rate between AVDM (3.62 ± 0.29Hz) and AVVL (3.32 ± 0.19Hz = −2.874, p = 0.68).

### Oscillatory entrainment of unit activity by the local LFP theta

**Figure 10:**
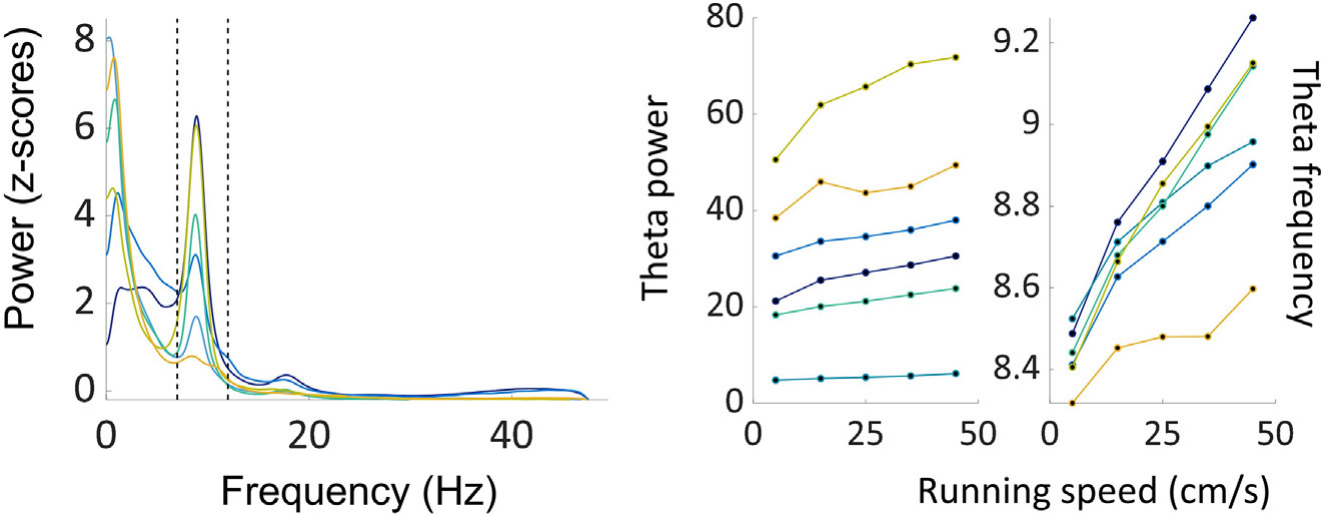
Theta oscillations locally recorded in anteroventral thalamic nucleus (AV) are modulated by running speed Left, average normalized power spectrum across all trials, color coded by animal (n = 6). Dotted lines define theta range (6-12Hz). Right, average speed-power and speed-frequency relationships across all trials for each animal, color coded as in A.

We examined oscillatory activity in the local LFP, as a prelude to looking at theta modulation of spiking and found notable activity in the type-1 theta frequency range (6-12Hz; average power: 3.49 ± 2.53; average theta-delta ratio: 1.32 ± 1.65; average frequency: 8.79 ± 0.64Hz). Across all six animals, we found a strong modulation by the animals’ running speed (Figure 10; r = 0.90 ± 0.13 for theta power and 0.87 ± 0.18 for theta frequency).

We next analyzed the theta-band properties of single cells. Theta rhythmicity and coupling were assessed by computing an index of rhythmicity (IR) and coupling (IC), respectively (Figure S6), and then we looked at two other known theta-associated phenomena: theta phase precession and theta-skipping.

Theta-by-HD cells, place cells, and theta non-directional cells were theta phase-locked, with the same average IC and IR, all being significantly higher than non-theta-rhythmic HD cells (IC; KW test; H(3) = 206.54; IR; H(3) = 180.86; all p < 0.001; Table 3). We also looked at a phenomenon known as theta-skipping, in which the autocorrelogram reveals a second theta peak larger than the first, due to a subpopulation of cells that fire on alternate theta cycles.^51^ We saw theta-skipping in 29.11%, n = 46/158) of Theta-by-HD cells (Figure S7).

**Figure 11:**
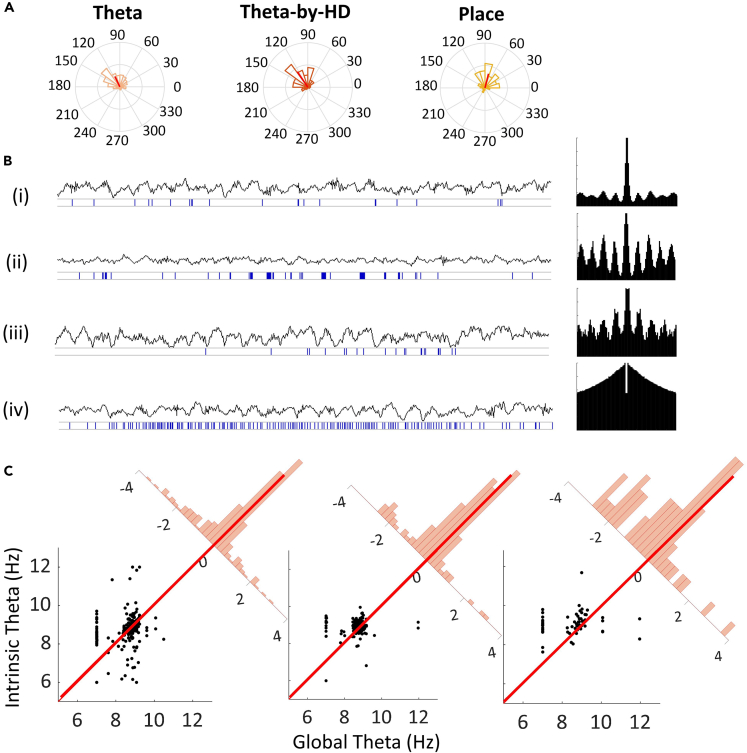
Periodic firing of anteroventral thalamic nucleus (AV) units during theta states (A) Circular histograms of preferred theta phases for theta-modulated cells: theta non-directional (n = 285), theta-by-head direction (HD: n = 158), place (n = 61). Red line is the R-vector pointing toward the mean preferred theta phase. Radius = 0.3. All cells fire between 0 and 180° (descending theta phases) but place cells fire slightly earlier, just after the peak (closer to 0). No difference between firing phases of theta and Theta-by-HD cells. (B) Examples of unit recordings and raw local field potential (LFP) trace recorded from the same electrode (for 3s of recordings). Cells are in the following order: (i) Theta non-directional; (ii) Theta-by-HD; (iii) Place; (iv) HD non-theta cells. The corresponding temporal autocorrelogram for each cell, between ±500ms, is shown to the right. Theta cells with no spatial properties mostly fired single spikes (not part of a train) while Theta-by-HD and place cells discharged rhythmically grouped spike trains (groups of 2–3 spikes with short inter-spike intervals) at theta frequencies. Theta-modulation is not visually detectable in the traces of non-rhythmic HD cells. Place cells show theta phase precession: spikes occurred slightly earlier with successive theta cycles. Note theta-skipping for the Theta-by-HD cell. (C) Analysis of theta phase precession for the phase-locked units. Intrinsic vs. global theta frequency (Hz), shown as scatterplots in the same order as in A. Each marker is a cell; if a marker is above the diagonal line, the cell was phase precessing. Corner histograms display the distance of each marker from the diagonal. x axis, distance to diagonal (a negative distance means the cell falls above the diagonal). Place and Theta-by-HD cells: distributions are toward negative values. Theta non-directional cells: more centrally aligned (weaker phase precession).

For cells that fired locked to the local LFP theta (which is phase-coupled and precedes hippocampal theta by 7-10ms^26^), we computed the preferred theta phase and plotted these values in circular histograms (Figure 11A). For all cells, spikes were aligned to descending theta phases. The theta peak was defined as 0° and place cells fired closer to the peak (76.02°; R-vector = 0.59; Rayleigh test, z = 21.65, p < 0.0001) while Theta-by-HD cells (124.02°; R-vector = 0.67; z = 69.82; p < 0.0001) and theta non-directional cells (115.42°; R-vector = 0.50; z = 71.58) fired slightly after place cells, closer to the theta trough. Both differed significantly from place cells (two-samples WW tests, Theta-by-HD vs. theta, *F* = 1.96; p = 0.16; Thteta-by-HD vs. place, *F* = 31.48; theta vs. place, *F* = 18.15; all p < 0.0001), suggesting sequential, phase-offset activation between these cells. The earlier average phase preference for place cells might be due to the steeper precession of firing phases relative to theta (shown below).

For the theta-modulated neurons, we also looked at the phenomenon of phase precession, first reported in place cells,^52^ in which successive spikes from a bursting neuron occur at progressively earlier phases of the simultaneously recorded theta LFP. This can be revealed if average intrinsic theta frequency of a neuron is slightly faster than global theta (Figures 11B and 11C). Comparing intrinsic oscillation frequency to the LFP frequency allows us to derive a convenient count of precessing neurons. We found evidence of phase precession in place cells (intrinsic vs. global frequency: 9.09 vs. 8.54Hz; two-sample t-test, *t*(60) = −3.60, SD = 1.20, p < 0.0001) and Theta-by-HD cells (intrinsic vs. global frequency: 8.9 vs. 8.63Hz; *t*(157) = −4.08, SD = 0.82, p < 0.0001). Theta cells did not show strong phase precession (intrinsic vs. global frequency: 8.76 vs. 8.64Hz; *t*(284) = −2.46, SD = 0.87, p = 0.01). Overall, 77.05% (47/61) place cells and 63.92% (101/158) Theta-by-HD cells phase precessed. Proportions did not differ from each other (χ^2^ = 3.46, p = 0.063), but were higher than the theta cell proportion (Theta-by-HD vs. Theta, χ^2^ = 4.96, p = 0.026; place vs. Theta, χ^2^ = 11.89, p < 0.0001).

We finally compared between the two AV subfields. Previously, we found that theta activity differed between the two RSC subregions, being more present in gRSC than dRSC.^16^ In contrast, there was no theta difference between AV subfields (AVDM vs. AVVL; two-sample WRS tests, IC: 0.17 ± 0.01 vs. 0.14 ± 0.01, z = 0.21, p = 0.834; TS index: −0.004 ± 0.01 vs. −0.007 ± 0.01, z = 0.05, p = 0.960).

### Velocity sensitivity and anticipatory firing

**Figure 12:**
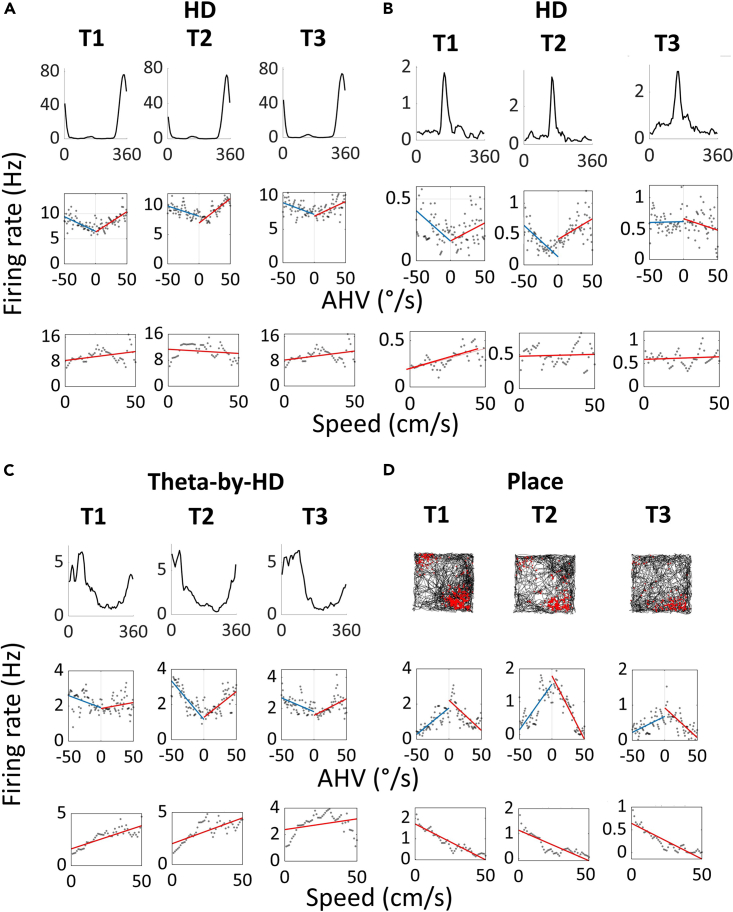
Spatial cells are modulated by linear and angular head movements (A–D) Examples of (A-B) non-rhythmic head direction (HD), (C) Theta-by-HD and (D) place cells showing conjunctive modulation by running speed and angular head velocity (AHV). Each cell was recorded across three consecutive trials. Trial type is at the top (T1, T2, T3; T2 was in darkness). For each cell, the following plots are shown, from left to right: tuning curve of firing rate (Hz) vs. HD (degrees) or spikemap for the place cell; scatterplot of instantaneous firing rate (Hz) vs. AHV in 2°/s bins; scatterplot of instantaneous firing rate vs. running speed in 2 cm/s bins. Linear regression lines were fitted to derive the slope and the y-intercept of the rate-AHV (clockwise (CW), red; counter-clockwise (CCW), blue) and the rate-running speed relationships. All conjunctive cells displayed the same direction of linear and angular activation (both positive or both negative).

We analyzed movement correlates, in linear and angular domains. Most of the cells showed positive responses to speed (n = 194/269; 72.12%) and AHV (n = 32/44; 72.73%), that is firing rates increased as the animal ran faster, and its head turned faster in both turning directions (see example of a negatively tuned place cell in Figure 12). We first look at linear, then angular speed correlates. Most cells (269/341; 78.89%) met the criterion for linear speed tuning. All cell types were different from zero (one-sample WSR test; non-rhythmic HD, z = 9.59; Theta-by-HD, z = 10.90; place cells, z = 6.79, all p < 0.0001). Equal proportions of speed-tuned units were observed in each cell group (Chi-squared tests; all p > 0.1; Table 3) and tuning strength did not differ between groups (average absolute s-scores: non-rhythmic HD, 0.56 ± 0.02; Theta-by-HD, 0.52 ± 0.02; place cell, 0.55 ± 0.03; KW test; H(2) = 1.771, p = 0.41).

In the angular domain, likewise, equal proportions of AHV-tuned units were observed in each group (44/341; 12.90%; Chi-squared tests; all p > 0.1; Table 3) and strength of AHV activation did not differ across cell types (average absolute AHV scores: non-rhythmic HD, 0.22 ± 0.01; Theta-by-HD, 0.24 ± 0.01; place cell, 0.25 ± 0.02; KW test; H(2) = 2.31, p = 0.315) and differed slightly but significantly from zero (one-sample WSR test; non-rhythmic HD, z = 9.58; Theta-by-HD, z = 10.90; place cell, z = 6.791, all p < 0.0001), suggesting weak, yet significant, AHV activation. For almost all AHV-tuned cells (42/44; 95.45%), firing rate modulation was symmetric (no effect of turning direction; paired-sample WSR test, z = 2.06, p = 0.04). Most angular-tuned neurons were conjunctively tuned to linear speed (n = 39/44; 88.64%).

We then compared across AV subfields: proportions of cells responding to speed or AHV did not differ between AVDM and AVVL (see values Table 3; all p > 0.05).

**Figure 13:**
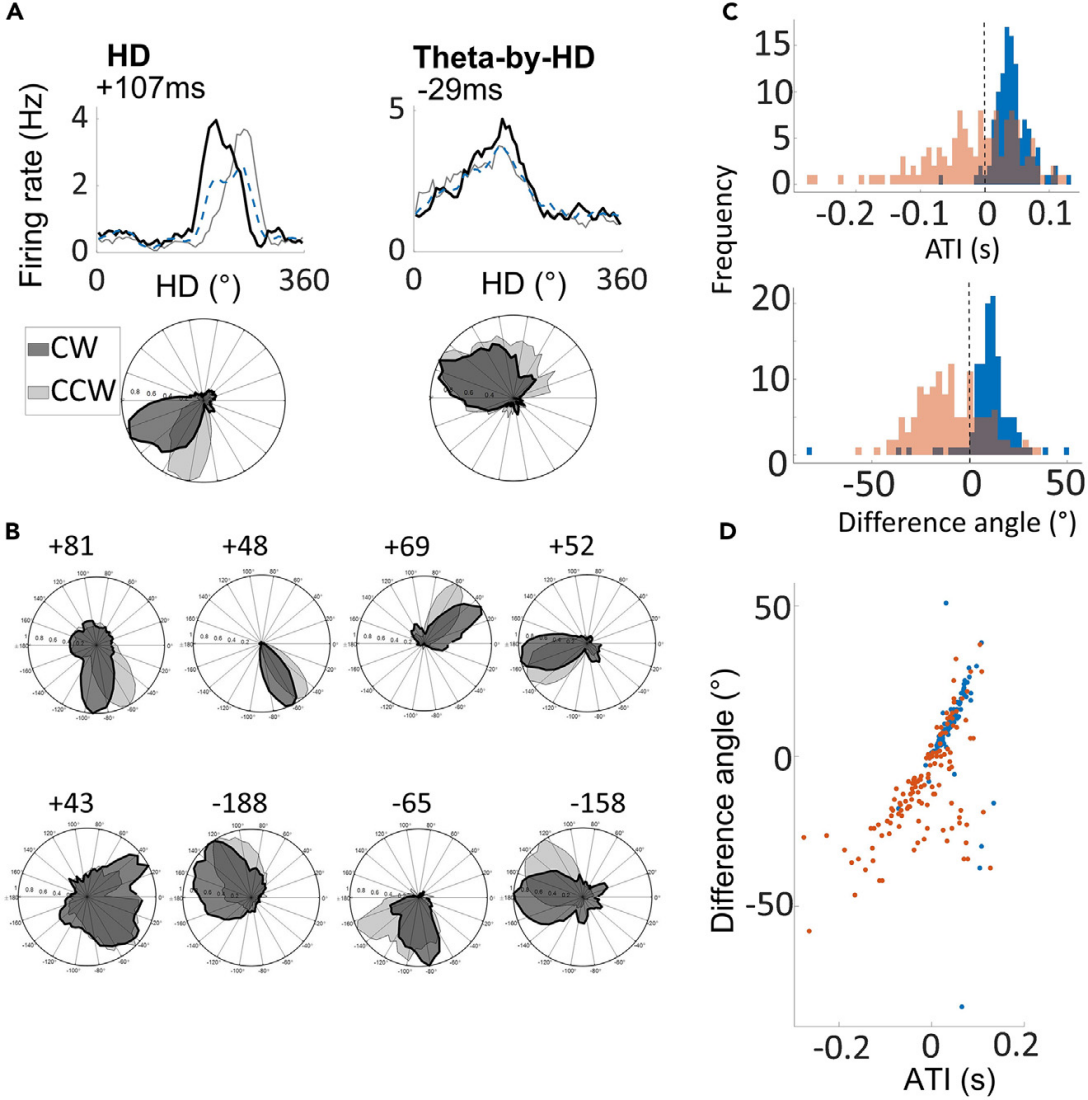
Non-rhythmic head direction (HD) cells are more anticipatory than Theta-by-HD cells Tuning curves decomposition analysis used to derive a cell’s anticipatory time interval (ATI). (A) Tuning curve decomposition analysis for a classic HD (non-rhythmic) and a Theta-by-HD cell. For each cell, the tuning curve of firing rate (Hz) vs. head direction (HD; degrees) is shown as smoothed histogram (top) and as polar plot normalized to 1Hz (bottom). Tuning curves were decomposed into clockwise (CW; black) and counter-clockwise (CCW; gray). The standard tuning function (blue) includes all spikes, including those emitted during immobility. ATI for each cell is reported. (B) More examples of the cells in A. Theta-by-HD cells are less anticipatory (smaller CW-CCW angular separation) and maintain their broader tuning curves also when decomposed into CW and CCW (see analysis in supplemental information and Table 5). Note that negative ATIs mean tuning functions shift in opposite directions during CW and CCW turns compared to positive ones. (C) Frequency distribution of ATIs and CCW-CW difference angles, compared between classic HD (blue) and Theta-by-HD cells (red). For both measures, Theta-by-HD show a greater variability while classic HD cells are skewed toward positive values. (D) Positive correlation between these measures. Markers represent cells, color coded as in C. The more a cell is anticipatory, the more the tuning curve shifts in opposite directions during CW and CCW turns.

To conclude, we looked at anticipatory firing. HD cells can fire in relation to future, rather than present, HD by integrating information about current heading direction with the velocity at which the head is turning.^53^ Non-rhythmic HD cells were more anticipatory (anticipatory time interval (ATI) = 41.7 ± 2.56ms), while Theta-by-HD cells exhibited greater variability (−17.3 ± 6.64ms; Figures 13A and 13B). ATIs were positive for 96% of non-rhythmic HD (107/112) and for 56% of theta-by-HD cells (59/128). ATI values differed from each other (two-sample WRS test, z = 7.05, p < 0.0001; Figure 13C). This time delay suggests that Theta-by-HD cells may arise from the output of several non-rhythmic HD cells with similar PFDs, which could also explain their broader tuning curves.

**Table 5:** Analysis of tuning curve shape for clockwise (CW) and counter clockwise (CCW) turns, and standard

	CCW	CW	Standard
Tuning width (degrees)			
Non-rhythmic HD	93.29 ± 3.03	95.1 ± 2.99	92.08 ± 3.080
Theta-by-HD	131.53 ± 1.32	131.5 ± 1.23	130.81 ± 1.29
Peak rate (Hz)			
Non-rhythmic HD	28.22 ± 3.05	28.57 ± 3.15	26.49 ± 2.91
Theta-by-HD	5.81 ± 0.38	5.96 ± 0.40	5.34 ± 0.35

HD = head direction cells.

CCW-CW difference angles were positive for non-rhythmic HD (9.71 ± 1.32°) but negative for Theta-by-HD cells (−9.54 ± 1.54; Kuiper two-sample test, k = 9312, p = 0.001). Non-rhythmic HD cell, but not Theta-by-HD cell values, differed significantly from zero, in line with the greater Theta-by-HD cell variability (one-sample WSR test; HD, z = 8.85, p < 0.0001; Theta-by-HD, z = −1.82, p = 0.069). Implant site cannot explain this variability: correlation between implant location and average ATI values did not reach significance (Pearson’s *r* = −0.01, p = 0.87), and there was no difference in the proportion of Theta-by-HD cells with negative ATIs between the two subfields: AVDM and AVVL (Chi-square test for differences in proportion; χ^2^ = 0.24, p = 0.625). The depth of the cell within AV may also be a contributing factor. As expected, there was a positive correlation between ATI and difference angle measures (Pearson’s r = 0.63, p < 0.0001; Figure 12D).

## Discussion

We investigated the single neuron processing of spatial, directional, temporal, and movement parameters in the two anteroventral thalamic (AV) subfields: dorsomedial (AVDM) and ventrolateral (AVVL). These experiments were motivated by recent findings that these two subfields project differently to granular and dysgranular RSC subregions (gRSC and dRSC) and that each RSC subregion has distinct directional and theta signaling properties.^14^^,^^16^ Our first novel finding is that we identified a population of place cells, with properties like those of hippocampal place cells but with different firing field metrics. The second is that AV cells, including place cells, came in two waveforms: broad and narrow, although these did not conform to the spiking classes (constituting another difference from hippocampal recordings^54^^,^^55^^,^^56^^,^^57^). Further, we compared these properties between AVDM and AVVL and found differences in the extent to which theta and directional information is combined at the single cell level. Specifically, we replicated previous AV neuronal response findings from Tsanov and colleagues^26^ that identified two types of signals: a theta signal, reflected in the burst firing of neurons at 7-12Hz frequencies, and a directional signal reflected in head-direction (HD)-specific firing. These two signals interacted to produce three types of neuronal activity: theta-modulated but non-directional cells, directional but non-theta-modulated cells, and a mixed subtype (in the Theta-by-HD cells) expressing both types of neuronal activity. We discuss these findings and their implications in more detail below and relate them to our recent neuroanatomical results that have demonstrated two distinct thalamocortical pathways between AV and RSC.^16^

### Spatial tuning

The most notable finding from this experiment was the observation of a very clear spatial signal, manifest as the occurrence of “place cells”—that is, cells that fired in a restricted region of the environment. These cells resembled classic hippocampal place cells recorded in CA1 in several respects, and indeed when we first observed them, we thought they might be hippocampal cells, since the dorsal-most part of the hippocampus overlies the anterior thalamus. However, careful examination of the histological slides did not reveal any evidence of explantation of hippocampal tissue into the AV. Also, spatial firing was observed throughout the dorsoventral extent of the electrode’s passage through the AV thalamus over the course of many sessions of recording (and indeed was more pronounced ventrally), and in most cases, co-occurred with recordings from Theta-by-HD cells, which do not occur in hippocampus.^58^^,^^59^

We compared these place cell responses with those of hippocampal place cells recorded in a similar-sized environment,^47^ which indicated that AV place fields were smaller and more spatially compact than have been reported for CA1 place cells.^47^^,^^55^^,^^56^

The credibility of this observation is further supported by the fact that place cells have been reported in other thalamic nuclei: the adjacent anteromedial thalamic nucleus (AM),^60^ the nucleus reuniens,^61^ and the paratenial nucleus.^60^ Until now, the literature lacked a specific description of the AV nucleus. It is unlikely that our electrodes penetrated AM: they were more lateral, and the co-occurrence of Theta-by-HD cells was not reported in AM.^60^ However, the AV place cells resembled AM place cells in other respects, such as the compact nature of the place fields and narrow waveforms. The findings of place cells in several thalamic nuclei, as well as in the anterior claustrum^62^ invites a more detailed investigation of whether the thalamus is a source or recipient of hippocampal place information, of how the signal is mixed with other signals such as HD and theta, and of what this processing might be for (see below).

### Directional tuning

We then examined the sub-class of neurons that showed directional tuning. These fell into two distinct categories: theta-modulated with broad tuning, and non-theta-modulated with narrow tuning. The latter resembled cells typically seen within the main HD circuit linked to the AD,^8^ including in their tendency to anticipate the animal’s head direction by a small amount. By contrast, Theta-by-HD cells showed reduced directional tuning and occasionally (n = 20; 13%) showed some conjunctive spatial tuning, which was much weaker than in place cells, and did not show anticipatory firing. Results indicate the presence in AV of two separate HD networks: a global HD network, possibly inherited from corticothalamic projections, and a theta-modulated HD network that may receive more indirect, or weaker, inputs from the main HD system, i.e., from non-rhythmic HD cells within AV.

### Cell-type heterogeneity

We observed that physiological heterogeneity of neurons in the AV was accompanied by heterogeneity in waveform shape, temporal firing patterns, directional firing patterns, and spatial firing, so we examined whether these physiological and functional properties were correlated. In terms of waveform shape, we found two distinct clusters, reflecting a narrow spike and broad spike, with the broad type being more prevalent in AVDM. This is consistent with their respective cell morphology; magnocellular (large) cells are prevalent in the AVDM, while more parvocellular (small) cells are in the AVVL.^30^^,^^63^ We did not, however, find functional correlates of these differences. In terms of temporal firing properties, place cells and Theta-by-HD cells fired rhythmic spike trains at theta frequencies, with or without bursting, while non-rhythmic HD cells only fired single spikes unrelated to theta. For the spatially modulated neurons, namely Theta-by-HD, place, and non-rhythmic HD cells, we did not find any waveform correlates, with around half of neurons in each functional type being broad, and the other half, narrow. Place modulation was equally represented in these two categories. All theta-modulated cells preferentially fired on descending theta phases. Place and Theta-by-HD cells showed firing phase precession in time, which was steeper than for the non-directional theta units. These functional cell types were differently distributed between AVVL and AVDM. Altogether, there are a range of physiologically distinct cell types in AV, but they do not conform clearly to functional categories.

### How do AV cells acquire their specific response properties and what are they for?

Directional modulation in AV is likely to be driven by corticothalamic input from the main HD network via the RSC (in turn inherited from the subcortical HD ring attractor^64^). The AV may inherit theta and spatial modulation from the septo-hippocampal spatial system, directly^65^ or via the MMN (Figure 1). Future studies are needed to test the effects on AV cell populations of reversible inactivation of their subcortical vs. cortical/septal inputs.

The main outputs of AV are to RSC, subicular complex and hippocampal formation.^17^^,^^18^^,^^19^^,^^20^^,^^44^^,^^66^ An intact ATN is required to establish place cell firing in subiculum but not in CA1.^67^^,^^68^ Moreover, place modulation in dRSC is only partially disrupted by bilateral, but not unilateral, dorsal CA1 lesions.^69^ Given our findings, these effects could be mediated by mechanisms independent of hippocampus, possibly by direct AV place cell discharge to RSC and subiculum.^5^^,^^17^^,^^30^^,^^70^^,^^71^ Interestingly, evidence shows that AV axons in dRSC carry spatial information.^72^

### The two AV-RSC pathways are functionally distinct

We now come back to one of the primary motivations for this investigation, which is to see whether some of the differential directional properties occurring in gRSC vs. dRSC might be accounted for by differences in their AV afferents. Based on our previous anatomical study showing differences in AV to RSC connectivity,^16^ we compared the properties of neurons recorded in AVDM vs. AVVL. We found no difference in either theta modulation or directional modulation. However, what differed was the conjunction between these signals, since AVVL had more conjunctive neurons. We found the following differences (summarized in Table 3): (1) classic HD (non-rhythmic) cells were more prevalent in AVDM (21% vs. 13%); (2) classic theta cells were also more prevalent in AVDM (45% vs. 34%); (3) Theta-by-HD cells were prevalent in AVVL (26% vs. 16%); (4) place cells were also more prevalent in AVVL (11% vs. 5%).

These differences suggest that the AV-RSC pathway is not homogeneous but may comprise two distinct inputs, differing in the extent to which theta and spatial signals are integrated. One input (AVDM to gRSC) is more involved in the relay of theta and HD signals. The other input (AVVL to dRSC) is more involved in the integration of theta and spatial signals, namely head orientation and spatial location. This fits with the existence of a wider “tripartite system” for spatial and memory functions,^9^ posing the existence of parallel cortical/hippocampal and subcortical/ATN memory systems acting together on shared cortical areas, like the RSC.

It will be important in future studies to test coupling of AV-RSC activity by means of simultaneous recordings, or after selective inactivation of the AV plus recordings in RSC. AVDM and AVVL are also differentially connected to the subicular complex, medial prefrontal cortex, and secondary motor cortex.^38^^,^^73^ The specific contribution of AV to neuronal firing in these structures remains to be determined. However, evidence shows that permanent or temporary manipulations to the AV alone, or in combination with the AD and AM, disrupt navigation and episodic memory processes in rats and macaque monkeys.^2^^,^^6^^,^^68^^,^^73^^,^^74^^,^^75^^,^^76^^,^^77^^,^^78^^,^^79^^,^^80^^,^^81^ Furthermore, manipulations to the ATN affect spatial responses in interconnected structures including the hippocampus, entorhinal cortex, and subicular complex,^7^^,^^21^^,^^67^^,^^68^ and reduce the expression of functional gene markers in RSC^79^^,^^82^^,^^83^ to a greater extent than mammillothalamic tract damage.^79^

### Conclusion

Our work addressed an important gap in our understanding of the spatial, and therefore cognitive functions of the ATN. Formerly regarded as providing a relay to, or diffuse arousal of, cortical temporal structures, the stability and precision of the spatial signals in our recordings emphasizes a cognitive role for the ATN, possibly forming a complementary system to the hippocampal formation. These results help to showcase how the brain uses spatial information earlier on, at the thalamic level, to construct an increasingly complex cognitive map, with the AV being a building block for this map.

### Limitations of the study

Our study used tetrodes, which provide stable recordings but a low yield of cells, meaning that results needed to be compiled over a great number of animals and recording sessions. Additionally, because of the need to advance the electrodes between sessions, the precise location of each neuron could not be assessed directly but only reconstructed *post hoc* using brain histology together with documentation of the amount of electrode advancement. However, the consistency of findings between animals compensates for this to some extent. Future work making use of new silicon probe high density recording technology will be able to replicate these findings with a larger dataset.

We reported cells with locational selectivity that displayed place fields that were highly spatially localized, round and, in cases of multiple place fields, regularly arranged. Given the small size of our testing box (90 × 90cm), it may have been possible to misclassify grid cells as place cells, as we would only be able to detect small grid scales.^84^ While we have ruled out electrode-dragging issues and axonal recordings to explain our place cell results, evidence of synchronous firing in the spike cross-correlation between co-recorded place and Theta-by-HD cells would provide final indication that cells are monosynaptically connected. Although we did not find any evidence of such correlated activity, it is noteworthy that, even if place cells were part of the AV neuronal circuit rather than outsiders, chances of finding a place cell-non place cell pair that were co-recorded and shared one synapse would be low.

## STAR★Methods

### Key resources table

**Table:** 

REAGENT or RESOURCE	SOURCE	IDENTIFIER
**Experimental models: Organisms/strains**		

Lister Hooded rats	Charles River Laboratories	Crl:LE

**Software and algorithms**		

MATLAB version R2019a	The Mathworks, Inc.	https://www.mathworks.com/downloads/
TINT	Axona Ltd., UK	http://www.axona.com/

**Deposited data**		
Figshare	https://figshare.com/s/d2e540c7af8d308848f3	https://doi.org/10.6084/m9.figshare.22802861

**Other**		

DacqUSB	Axona Ltd., UK	http://www.axona.com/
Euthatal	Merial, UK	Vm 08327/4112
Leica DMR microscope	Leica Microsystems Wetzlar GmbH	http://www.leica.com

### Resource availability

#### Lead contact

Additional information and resource requests should be directed to the Lead Contact Kate Jeffery (Kate.Jeffery@glasgow.ac.uk).

#### Materials availability

This study did not generate new unique reagents or materials.

### Experimental model and study participant details

#### Study design and animals

Six adult (>3mo) male Lister Hooded rats (Charles River, UK) weighing between 420 and 480g at time of surgery were used for electrophysiology recordings. Prior to the implant surgery, rats were housed in groups of four and handled for up to 10 days and familiarized with transportation to the recording lab and open field square arena. Animals were housed singly post-implantation in transparent Plexiglas cages in a temperature and humidity-controlled environment (21°C). Ten days after recovery from surgery, food regulation started (20g/day/rat) to maintain 85-90% of free-feeding body weight and testing began. All animals had free access to water throughout all experiments. Testing was conducted in the light phase of a 12h light/dark cycle (lights on at 7am). All experiments were carried out in accordance with UK Animals (Scientific Procedures) Act, 1986 and EU directive (2010-63-EU), complying with ARRIVE guidelines for the care and use of laboratory animals.

#### Surgery

Standard stereotaxic surgery techniques for implantation of chronic recording electrodes were followed.^85^ Five animals were implanted in the left hemisphere and one in the right. Of these six animals, two were implanted in AVVL (R222 and R449) and four in AVDM (R762, R448, R651 and R652). In three of the AVDM animals (R448, R651 and R652), electrodes were advanced throughout the entire AV extent, sampling first AVDM and then moving into AVVL (see Table 1 for details on the cells recorded for all animals and AV subfields). AVDM implant coordinates were: AP, -1.7; ML, ± 1.4 − 1.5; DV, 3.6 (in mm from Bregma). AVVL implant coordinates were: AP, -1.7; ML, ± 1.7 − 1.8; DV, 3.4.

#### Behavioral protocol and testing apparatus

Animals were screened daily for single unit activity. Screening and testing sessions took place inside a 90x90cm square arena with 60cm high walls, within a cue-rich room. The walls and floor of the box were covered with black vinyl sheeting. Animals were transferred inside a black box to the experimental room from their homecage, and connected to the multichannel recording apparatus (DacqUSB, Axona). Each trial was initiated via remote control after placing the rat inside the arena at a pseudo-random location and orientation.

Animals were screened daily for single unit activity. Screenings were made in an open field box within a cue-rich room. Spikes were monitored and when a single unit was isolated using TINT software (Axona, UK), the rat was removed from the arena and transferred back to homecage. The arena’s floor and walls were cleaned, and the rat was placed back in the arena for the experimental session.

Each basic experimental session was composed of two trials (“Light 1”, “Light 2” trials), lasting 8 or 16 minutes depending on the animal, during which rats foraged for rice scattered inside the arena. Animals were never disoriented prior to being placed in the arena. In this way, we did not disrupt internal sources of spatial information.^86^ During each inter-trial interval, animals were removed from the arena and transferred back to the homecage, and the arena was cleaned to remove uncontrolled intra-maze non-visual cues. In some sessions, cells from four of the six implanted rats were additionally recorded in the same square box after switching off the room lights and switching on an infrared light (“Dark” trial), to assess the contribution of visual inputs to spatial firing patterns.

#### Video tracking and signal collection/processing

During recording, the position and head direction of the animal were obtained from video tracking of two light-emitting diodes (LEDs) on the headstage, one large and one small and separated by 7cm. The LEDs were imaged with an overhead camera at a 50Hz sampling rate. Simultaneously, single unit and local field potential (LFP) signals were sampled at 48kHz using a headstage amplifier plugged into the microdrive connector and routed to the multichannel recording system (DacqUSB, Axona, UK) via a flexible lightweight cable. For single units, the signal from each electrode was differentially recorded against the signal from an electrode on a different tetrode with low spiking activity. It was amplified 15-50k times and band-pass filtered from 300Hz to 7kHz. For LFP, the signal was collected single-ended (250Hz), amplified 5K times, filtered with a 500Hz low-pass filter and a 50Hz notch filter. Spike times, LFP signal, position in x- and y-coordinates and directional heading in degrees were saved for offline analysis.

### Quantification and statistical analysis

#### Data analysis

Data were analyzed offline using TINT (Axona Ltd) and MATLAB R2019a (MathWorks, USA) with custom-written software. The CircStat toolbox was used for circular statistics.^87^ Statistics are reported in the main text.

#### Spike sorting

Spike-sorting was performed using KlustaKwik^88^ followed by manual refinement using TINT. Single-unit inclusion criteria were (1) an average waveform displaying the classical action potential shape, and (2) fewer than 10% of spikes occurring in the first 2ms of the autocorrelogram (action potential refractory period). Only cells meeting these criteria were accepted into further analysis.

#### Waveform analysis

The width of the average spike waveform was used to separate narrow from broad spiking cell types^89^ using a clustering protocol proposed by Ardid et al.^90^ (open-source code from the public Git repository: https://bitbucket.org/sardid/waveformAnalysis). We fit the distribution of waveform widths with two Gaussian probability distributions. Two cutoffs were defined on these models as points at which the cumulative density function (CDF) for one Gaussian distribution was 10 times larger than the CDF for the other Gaussian i.e., the probability to belong to a group was 10 times larger than the probability to belong to the other one. Width values smaller than the first cutoff were classified as narrow, whereas values higher than the second cutoff were classified as broad. Intermediate waveforms were those falling into the dip of the bimodal distribution. The calibrated version of the Hartigan Dip Test discarded unimodality for the distribution (*p* < 0.01).

#### Theta analysis

The Fast-Fourier transform (FFT) was used to find the LFP power spectrum. To compare across trials and animals, power measures within each trial were normalized to z-scores and plotted and smoothed with a Gaussian kernel (bandwidth = 2Hz; standard deviation = 0.5Hz). Theta frequency power is the maximum z-score within the 6-12Hz theta frequency range. Average theta frequency is the frequency at peak theta power.

For phases analyses, the LFP signal was bandpass filtered at theta frequency range (6-12Hz) with a 4th order Butterworth filter. The Hilbert transform was applied to the bandpass-filtered LFP to derive instantaneous theta phases. Each theta cycle was defined such that peaks occurred at 0 and 360°, and troughs at 180°. To determine at which point to apply the Hilbert Transform, the LFP signal (Hz) was linearly interpolated with spike time (s), with each spike being assigned to the phase of the theta cycle at which it occurred. To visualize the spike-LFP relationship of a cell, firing phases were double-plotted in a frequency histogram. The probability distribution of spikes relative to theta phases was derived by smoothing the spike-phase histogram using a circular kernel density estimation (KDE) method, with an automatically selected bandwidth parameter using the plug-in rule of Taylor.^91^

To obtain the relationship to running speed, the Hilbert transform was applied to the Butterworth filtered LFP signal to derive instantaneous theta frequency and amplitude that matched with running speed information recorded by the camera (50Hz). Instantaneous running speed was calculated as the distance between two consecutive position points, divided by the time between them. To analyze the relationship between instantaneous theta power vs running speed, we binned speed data into 10cm/s bin, within a range of 0-50cm/s. Average theta power was computed for each bin, and a linear regression model was fitted to the data using the least-squares approach. The slope of the regression line and Pearson’s linear correlation coefficient (r) between speed bins and the corresponding power were derived. The same method was used to analyze the relationship between instantaneous theta frequency and speed.

Spike-LFP coherence and rhythmicity were assessed by computing an index of rhythmicity (IR) and an index of theta phase-coupling (IC). Rhythmicity refers to the frequency of the temporal modulation of the spike-time autocorrelogram at theta frequency range (6-12Hz), while coupling refers to the phase of theta at which spikes occurred. We used both measures because spikes may be emitted with a timing phase-locked to theta even if there is no overt autocorrelogram rhythmicity.^12^^,^^92^ Autocorrelograms of the spike trains were plotted between ± 500ms using 10ms bins, normalized to the maximum value and smoothed (20 bins boxcar). The IR was calculated as the difference between the expected theta modulation-trough (autocorrelogram value between 60-70ms) and the theta-modulation peak (autocorrelogram value between 120-130ms), divided by their sum. It takes values between -1 and 1.^93^ Cells were considered as theta modulated if they passed the 99^th^ percentile shuffle cutoff for IC and had an IR>=0.001.^26^^,^^27^ For phase-locked cells, the preferred theta phase was found as the circular mean of all the spike phases. For the IC, phases were binned between 0 and 360° with 6° bins and the IC was found as the mean vector length (Rayleigh vector, CircStat toolbox) of these angles.^94^^,^^95^

Units that fire locked to theta oscillations can fire on every theta cycle, or on alternate ones (theta-skipping).^51^ To derive the theta-skipping index (TS), a curve was fitted to the autocorrelogram (10ms bins between ± 400ms) using the equation in Brandon et al.^51^:

y (x) = [a_1_ (cos (ωx) + 1) + a_2_ (cos (0.5∗ωx) + 1) +b] ∗ *exp*(−|x|/τ_1_) + (c. ∗*exp*(−x^2^/τ_2_^2^))

with x=autocorrelogram bins, m=maximum autocorrelation value. Fit parameters were restricted to the following values: a_1_ = [0, m], a_2_ = [0, m], b = [0, m], c = [−m, m], ω = [10π, 18π], τ_1_ = [0, 5] and τ_2_ = [0, 0.05]. TS index, bound between -1 and 1, represents the difference between the height of the first and second peaks of the fitted curve, divided by the largest of the two. For this analysis, we selected only cells that passed our theta phase-locking criterion (IC >= 99^th^ percentile shuffling). Among these cells, a theta-skipping cell had to meet following criteria: (a) a good (R2 > 0.7) fit of the model parameters to the autocorrelogram; (b) a baseline theta power component in the autocorrelogram (IR >= 0.001), and (c) the first side peak of the autocorrelogram smaller than the second side peak (TS > 0.1). All cells were confirmed by visual inspection of their autocorrelogram.

The approach used to investigate phase precession consisted of comparing the intrinsic oscillation of cells (theta frequency in the temporal autocorrelogram) to the global theta oscillation frequency (theta frequency in the LFP). When cells phase precess, they fire progressively earlier with each successive theta cycle, so on average the theta frequency in their autocorrelogram is slightly faster than the global LFP frequency.^96^ To derive intrinsic theta frequency, a decomposing sine wave of frequency ω was then fitted to the autocorrelogram (10ms bins between ± 500ms) using the equation in Grieves et al.^95^:

y (t) = a ∗ (sin(2π ∗ ω ∗ x + (π/2)) + 1) + b). ∗ *exp*(−|x|/t_1_ ) + (c ∗ *exp*(−(x^2^)/t_2_^2^))

where t is the autocorrelogram time lag, and a-c, ω, and t_1-2_ were fit to the data using a non-linear least squares method (Matlab function *fit*). Parameters were restricted to the following values: ω = [6, 12], a-b = [0 Inf], c = [0, 0.8], t_1_-_2_ = [0, 0.05]. Intrinsic theta frequency corresponds to the fit parameter, ω.^59^

#### Directional tuning curves

Directionality in the firing of a cell was quantified by the mean vector length (R-vector) of the directional tuning curve. Spike and HD data were sorted into 60 bins of 6°. The mean firing rates per angular bin (Hz) were smoothed with a 5-bin (30°) smoothing kernel and polar-plotted. The parameters that were used to quantify tuning curve characteristics were the mean vector length, or Rayleigh vector (R-vector, CircStat toolbox), directional peak firing rate (Hz), PFD (directional bin associated with the peak firing rate), tuning width (two standard deviations from the circular mean direction (φ), and concentration parameter*, k*.^97^ Cells were considered as directionally modulated if they passed the 99^th^ percentile shuffle cutoff for R-vector. Directional information content in bits/spike was also computed following the method of Skaggs et al.,^98^ which computes the amount of spatial information carried by each spike.

To analyze drift, we quantified the amount of variability in the firing direction of a cell when it fired >50% of its peak firing rate for the trial. We took all spikes emitted at these times (from the spike-time histogram), plus spikes emitted before and after it up to an angular distance of +/-50°. This defines a full sweep of the head through the cell’s PFD. A selection of spikes based on each individual sweep rather than on a reference frame from the whole dataset meant that we did not need the PFD prior, which would bias our sampling of spikes in case of drift. For each full-sweep event, we computed the circular KDE of the HD, using an automatically selected bandwidth parameter k.^91^ The KDE method was used to avoid arbitrary decisions on binning and because distributional properties are more easily assessed compared to a histogram (assessed next). Since the reliability of the density estimate depends on the number of data points,^99^ only sweeps containing at least 5 spikes were considered.

For each trial, we found the average width of all the KDE peaks (width at half-height), and how much variability there was in the absolute direction of these peaks (SEM of peaks location) and compared between cell groups using two-sample T-tests. Doing so allowed us to test two different, but non-mutually exclusive, hypotheses to explain broader tuning curves of Theta-by-HD cells: (1) tuning curves are inherently broader due to the directional firing range being larger, or (2) they reflect a less stable directional signal, due to PFD shifting to a larger extent during the trial. If the former is correct, there should be little jitter in the KDE peaks (small SEM of peaks location) but average width of the KDEs should be large. If the latter is correct, there should be a larger variability in KDE peaks (larger SEM of KDEs location) but small mean peak width.

#### Self-motion correlates

We quantified the relationship between instantaneous firing rates and linear/angular speed of the head using a speed score (s-score) and an AHV score, respectively. The instantaneous firing rate of a cell was derived from the smoothed spike-time histogram, obtained using 20ms bins (coinciding to 50Hz sampling rate), smoothed with a 250ms-wide Gaussian kernel. Dividing the spike count per bin by 0.02 converts counts to firing rate for each position data point. Instantaneous running speed was measured as the distance between two consecutive position data points, divided by the time between them. Instantaneous firing rate was binned by running speed (2cm/s bins, between 2 and 50cm/s) and fitted by a linear regression model using the least-squares approach. The s-score is the Pearson’s linear correlation coefficient between instantaneous firing rate and speed bins.^100^ Speed-tuned cells were considered to be those with an absolute s-score above 0.3.

Instantaneous AHV was measured as the angular difference between two successive HD data points, divided by the time between them.^101^ HD measurements were interpolated between consecutive samples to provide an estimate of HD at each AHV point. A 2°/s bin width was used to bin instantaneous firing rate by AHV, between -100 and 100°/s was used. A scattergram of firing rate vs AHV was plotted, and linear regression lines were fitted to AHV values between -2 and -50°/s, and between 2 and 50°/s. Values between -2°/s and 2°/s were discarded, as periods in which the head was not turning. CW and CCW AHV scores are the Pearson’s correlation between instantaneous firing rate and AHV bins. To quantify AHV modulation irrespective of the turning direction, we first considered absolute AHV values. AHV-responsive cells have an absolute AHV score above 0.3. Taking the absolute values was necessary because HD cells can both increase and decrease firing rates with AHV in different turning directions,^93^^,^^102^ having slopes that are positive in one direction and negative in the other, which cancel if raw values are taken. We then considered CW and CCW AHV scores separately to classify cells as symmetric or asymmetric.

#### Directional tuning curve cross-correlation

To compare cross-trial stability between non-rhythmic HD and Theta-by-HD cells, we used a cross-correlation approach. Smoothed tuning curves for each pair of trial (Light-Light; Light-Dark) were circularly cross-correlated in 6° steps and Pearson’s correlation (r) was computed at each step. Correlation at 0° was used as a measure of cross-trial stability for each cell. The distribution of all correlations was compared to the distribution of correlations derived from cross correlating tuning curves in the first trial with shuffled tuning curves from the second trial (spikes time shifted 10,000 times relative to HD). The 95^th^ percentile value from the distribution of shuffled correlations was taken as the chance-level criteria against which to test the similarity of directional fields for each cell.

#### Spatial analysis

Locational ratemaps were calculated by binning position and spikes data in 2x2cm bins, smoothed separately using a weighted Gaussian kernel (smoothing factor = 5cm) such that data points closer to a bin’s center had more influence on that bin’s firing rate. Bins that were >10cm away from the current bin had no influence (weight=0) and were not included in the Gaussian smoothing process. The smoothed ratemap was generated by dividing the smoothed spikemap by the smoothed dwelltime map.^103^ The overall spike count divided by session duration gave the mean firing rate (Hz) of the cell. The locational peak firing rate (Hz) is the firing rate of the cell in the bin with the maximum average rate. CA1 place cells were taken from Casali et al.^47^ had to show a peak rate in the locational field exceeding 1 Hz.

#### Isolation of spatial and directional spiking

To isolate spatial and directional influences (which could become artefactually coupled as the animal explores the box), we used a maximum likelihood approach, known as the position-by-direction (pxd) correction.^48^^,^^104^ We used the code taken from Burgess et al.,^104^ available online from these authors. Similar numbers of bins were applied to locations and directions. There were 60 6° directional bins and 64 locational bins. No smoothing was applied. This provides better convergence of the pxd algorithm and ensures that direction and location were given equal weighting in finding the solution. This method was applied to all cells (n=1 non-rhythmic HD cell for which the algorithm did not converge upon a solution was discarded).

Corrected measures of locational and directional information content and sparsity were calculated according to the formulas in Skaggs et al.^98^ Thalamic units were classified as place cells based on high locational information and a low sparsity, computed as the percentage of the arena’s surface over which a cell fired. A place field was defined as a group of at least 9 adjoining pixels in which firing rate was at least 20% of the peak firing rate, and peak firing rate was at least 1Hz.

#### Cell classification

To generate control data, a shuffling procedure was used in which each cell’s spike train was circularly time-shifted 10,000 times by a random amount between 20s and the duration of the recording session minus 20s, relative to the behavioral or theta phase data. The relevant scores were recomputed each time and values were pooled together to derive a control distribution for theta and HD modulation. A 99^th^ percentile cutoff was used to determine significance levels for individual cells to be theta- or HD-modulated. Given that the R-vector measure is very sensitive to small amounts of unimodality, setting the threshold high ensured that only cells with clear theta and HD selectivity made it into the sample and that the classified cells would certainly be considered as theta or HD had they been recorded from a different brain region.

The shuffle procedure was then used for cell classification, in addition to several other criteria as follows. A cell was considered theta-modulated if it fired rhythmic spike trains (IR >= 0.001) locked to theta (IC >= 99^th^ shuffle percentile). A non-rhythmic HD cell was defined as a cell with significant directionality (directional peak firing rate > 1Hz; R-vector >= 99^th^ shuffle percentile) that was not also theta-modulated. A Theta-by-HD cell showed both theta and HD modulation. A place cell was a cell with significant locational modulation (mean firing rate > 0.1Hz but < 10Hz; locational peak firing rate > 1Hz; locational information content > 0.8 bits/spike; sparsity score < 0.2). All the place cells reported were also theta-modulated. Conservative thresholds were chosen intentionally to set a lower bound for the proportions of place cells in the AV and ensure that only units with high locational selectivity were included in the sample.

#### Cell-specific temporal firing characteristics

To investigate temporal firing properties, inter-spike interval (ISI) histograms were created by binning all ISIs in the range 0-250ms with 2ms bins. The peak ISI of a cell was taken as the centre of the histogram bin with the highest count. A cell was classified as bursting if peak ISI < 6ms. The burst index was defined as the ratio of spikes sharing ISI < 6ms to all spikes emitted by the cell in a trial.

#### Anticipatory time interval (ATI) analysis

The anticipatory time interval (ATI) of a cell was estimated based on cross-correlating clockwise (CW) and counter-clockwise (CCW) tuning functions for that cell.^101^ For each cell, CW and CCW spikes were identified according to the angular head velocity (AHV) at which the head was turning when a spike occurred. CW spikes occurred at AHV > 60°/s, CCW spikes occurred at AHV < -60°/s. Spikes emitted at times in which the head was not turning were removed. CW and CCW spikes were time-shifted in steps of 20ms, from 20 to 160ms (coinciding with 50Hz sampling frequency). For each step, CW and CCW tuning curves were plotted, normalized to 1Hz and circularly cross-correlated by rotating one relative to the other in steps of 6° to find the rotation angle that produced the maximal cross-correlation (Pearson’s r) between the two curves. Angles were plotted as a function of the corresponding time shift, and a linear regression model was fitted using the least squares method. The ATI is the time shift required to align CW and CCW functions. The difference angle is the rotation angle that yields the maximal cross-correlation between CCW and CW tuning functions for a time shift of 0ms. Cells with cross-correlation below 0.7 were removed (non-rhythmic HD, n = 10; Theta-by-HD, n = 30).

#### Histology

After completion of all recordings, rats were anesthetized with isoflurane and received an overdose of sodium pentobarbital (Euthatal, Merial, UK). Rats were perfused transcardially using saline (0.9% sodium chloride solution) followed by 10% formalin perfusion (10% formalin in 0.9% sodium chloride solution). The brains were removed and post fixed overnight in 10% formalin, transferred to a 25-30% sucrose solution (25-30% in 0.1M PBS) for a minimum of 48h for cryoprotection. Brains were sliced coronally into sections of 40μm thickness on a freezing microtome. Sections were mounted on glass slides. Tetrode locations were histologically verified from cresyl violet stained brain sections. Photomicrographs were captured using a camera mounted on a Leica DMR microscope (Leica Microsystems, UK) with a x1.6 objective.

#### Statistics

If assumption of normality was met, comparisons between two groups were conducted using independent-samples or paired-samples t-tests. Otherwise, we used non-parametric statistical tests and comparisons between groups were conducted using the two-sample Wilcoxon Rank-sum (WRS) tests and paired-sample Wilcoxon Signed rank (WSR) tests. For more than two groups, a Kruskal-Wallis (KW) test was conducted, followed by multiple pairwise comparisons of group medians (SPSS, Bonferroni correction). A Chi-square test tested differences in the observed number of cells between two groups against an expected equal proportion. Two-sample Kolmogorov-Smirnov test (KS test) tested whether two cumulative density functions (cdf) differed significantly.

For circular data, a Watson Williams (WW) multi-sample F-test compared circular means between two or more groups. Equality of the two circular distributions was tested using a Kuiper test,^99^ and MATLAB functions *circ_ktest* for differences in concentration parameters. All statistical tests are two-tailed. In all figures and tables, *p*-value significance level: ∗=0.05 significance level, ∗∗=0.01, ∗∗∗=0.001.

## Data Availability

• All original data and code have been deposited at Figshare and are publicly available as of the date of publication. DOIs are listed in the key resources table. • Any additional information required to reanalyze the data reported in this paper is available from the lead contact upon request.
